# Supplementary material for: Eradicating the large white butterfly from New Zealand eliminates a threat to endemic Brassicaceae
Source: PLoS One. 2020 Aug 6;15(8):e0236791. doi: 10.1371/journal.pone.0236791 (PMC7410255; doi:10.1371/journal.pone.0236791)
Supplement: S1 Text — (DOCX) [file pone.0236791.s001.docx]

## S1 Text. Additional management details for the New Zealand *Pieris brassicae* eradication program.

The *Pieris brassicae* eradication program used a Coordinated Incident Management System framework to structure roles and responsibilities [1]. Roles and responsibilities included: Governance (a senior DOC manager who maintained an overview of the program, managed conflict and ensured resources remained available to the program); Project Manager (directed strategic and operational aspects of the work); Technical Advice Group (supported the program with advice on strategy, tactics and science); Operations Manager (directed daily work of field teams); Planning Manager (identified priorities for resource allocation); Logistics/Administration Manager (coordinated resources and ensured organisational needs were met); Public Relations Coordinator (transferred information to the public and stakeholders); and Data Manager (ensured data was captured, stored and analysed in a timely way to support decision making).

The Technical Advisory Group comprised three animal pest technical advisors from DOC including an entomologist, two entomologists from two government research institutes, and a private consulting entomologist.

DOC’s review of the eradication program was conducted by two staff with significant experience of other eradication programs, and one was a member of the TAG and had detailed knowledge of the attempt to eradicate *P. brassicae* [2]. Participants in the second review conducted by MPI included three TAG members, nine independent experts, and five MPI staff [3]. Prior to the review, participants were sent a report describing program progress [4].

References

1. New Zealand Government. The New Zealand Coordinated Incident Management System (CIMS). Wellington; 2014 Apr p. 68. Report No.: 2nd edition. Available: https://www.civildefence.govt.nz/assets/Uploads/publications/CIMS-2nd-edition.pdf

2. Briden K, Broome K. Great White Butterfly Review. Nelson: Department of Conservation; 2013 Aug p. 16. Report No.: DOCDM 1278224.

3. Gill G. Agenda: MPI & External Technical Advisory Group (Great White Butterfly) Technical Advisory Group Meeting. Wellington: Ministry for Primary Industries; 2013 Dec p. 2.

4. Phillips C, Brown K, Green C, Walker G, Broome K, Vander Lee B, et al. Great White Butterfly Interim Report Prepared for Ministry for Primary Industries External Technical Advisory Group, December 2013. Nelson: Department of Conservation; 2013 Nov p. 31. Report No.: docDM-1307089.
