# Supplementary material for: Eradicating the large white butterfly from New Zealand eliminates a threat to endemic Brassicaceae
Source: PLoS One. 2020 Aug 6;15(8):e0236791. doi: 10.1371/journal.pone.0236791 (PMC7410255; doi:10.1371/journal.pone.0236791)
Supplement: S1 Fig — (PDF) [file pone.0236791.s002.pdf]

Management blocks used by New Zealand's Department of Conservation in Nelson during its programme to eradicate *Pieris brassicae*

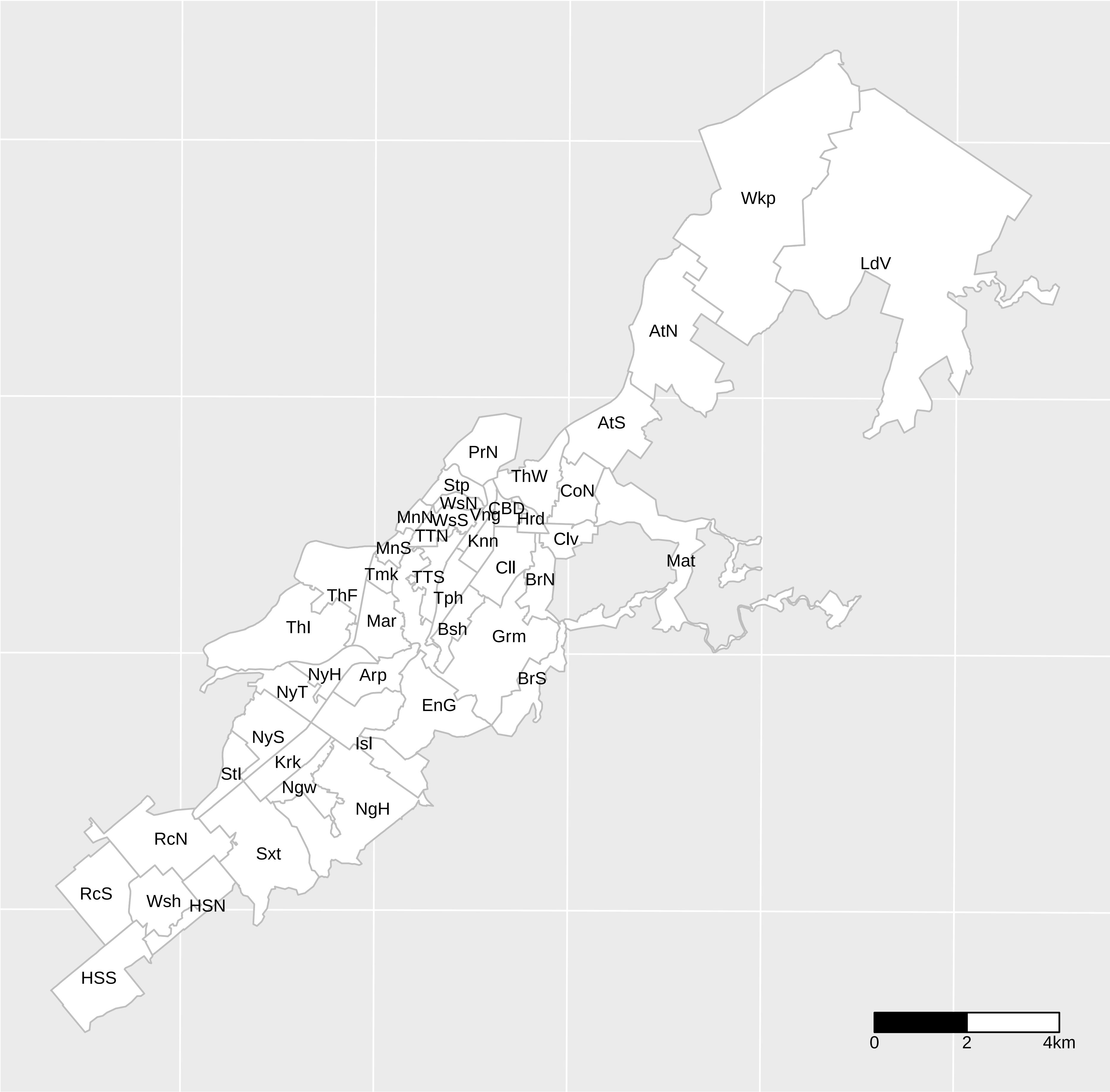

Code	Block
Arp	Arapiki
AtN	Atawhai North
AtS	Atawhai South
Bsh	Bishopdale
BrN	Brook North
BrS	Brook South
CBD	CBD
CoN	Centre of NZ
Clv	Cleveland
Cll	Colleges
EnG	Enner Glynn
Grm	Grampians
Hrd	Hardy
HSN	Hill Street North
HSS	Hill Street South
Isl	Isel
Krk	Karaka
Knn	Konini
LdV	Lud Valley
Mar	Maire
Mat	Maitai
MnN	Moana North
MnS	Moana South

Code	Block
NyH	Nayland Head
NyS	Nayland South
NyT	Nayland Tail
Ngw	Ngawhatu
NgH	Ngawhatu Hills
PrN	Port Nelson
RcN	Richmond North
RcS	Richmond South
Sxt	Saxton
Stp	Stepneyville
StI	Stoke Industrial
ThF	Tahuna Flats
ThI	Tahuna Industrial
Tmk	Tamaki
ThW	The Wood
Tph	Tipahi
TTN	Toi Toi North
TTS	Toi Toi South
Vng	Vanguard
Wkp	Wakapuaka
Wsh	Washbourne
WsN	Washington North
WsS	Washington South
