# Supplementary figures and images for: Eradicating the large white butterfly from New Zealand eliminates a threat to endemic Brassicaceae
Source: PLoS One. 2020 Aug 6;15(8):e0236791. doi: 10.1371/journal.pone.0236791 (PMC7410255; doi:10.1371/journal.pone.0236791)

## Additional Information 3. *Pieris brassicae* data record sheet.


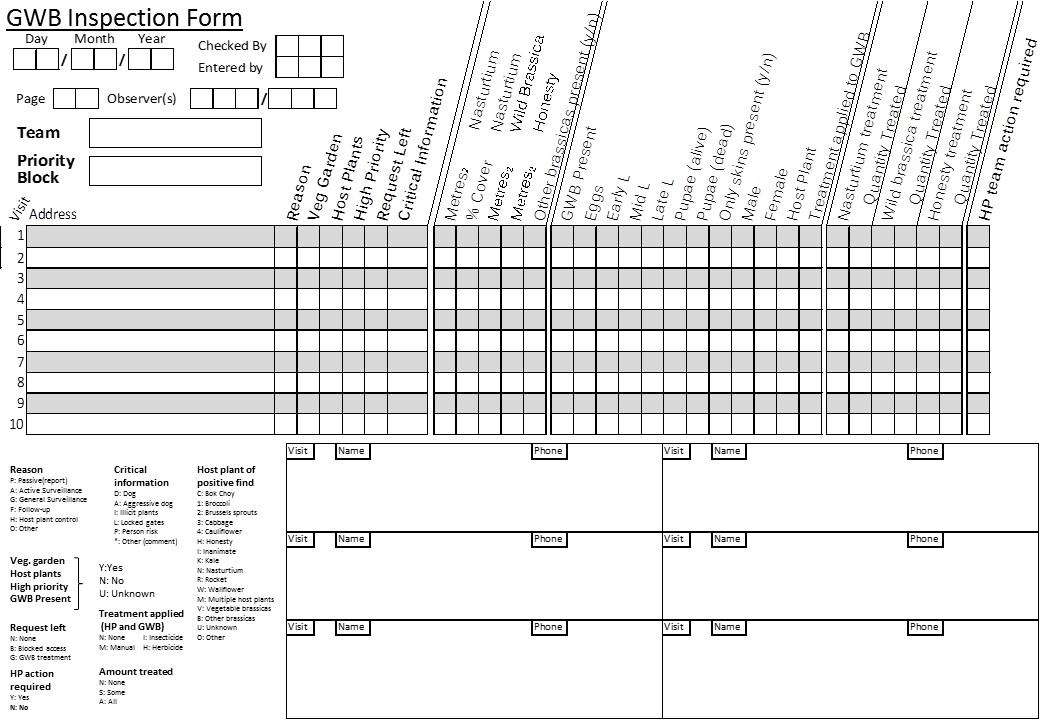

Supplement: S2 Fig — (DOCX) [file pone.0236791.s003.docx]
