# Supplementary material for: Eradicating the large white butterfly from New Zealand eliminates a threat to endemic Brassicaceae
Source: PLoS One. 2020 Aug 6;15(8):e0236791. doi: 10.1371/journal.pone.0236791 (PMC7410255; doi:10.1371/journal.pone.0236791)
Supplement: Attachment — Submitted filename: PONE-D-19-32860_reviewer (1).pdf [file pone.0236791.s005.pdf]

PLOS ONE

Eradicating large white butterfly from New Zealand eliminates a threat to endemic Brassicaceae

--Manuscript Draft--

**Manuscript Number:**	PONE-D-19-32860
**Article Type:**	Research Article
**Full Title:**	Eradicating large white butterfly from New Zealand eliminates a threat to endemic Brassicaceae
**Short Title:**	Insect eradication to help protect threatened endemic plants
**Corresponding Author:**	Craig Phillips, PhD<br>AgResearch Ltd<br>Lincoln, Canterbury NEW ZEALAND
**Keywords:**	invasion; invasive; non-native; alien; pest; insect; biodiversity threat; biodiversity conservation; environmental impact; native plant; endemic plant; Urban; community engagement; public awareness; social license; sightings; host plant destruction; biological control; chemical control; lure
**Abstract:**	In May 2010 the large white butterfly, Pieris brassicae L. (Lepidoptera: Pieridae), was discovered to have established in New Zealand. It is a Palearctic species that—due to its wide host plant range within the Brassicaceae—was regarded as a risk to New Zealand's native brassicas. New Zealand has 86 native species of Brassicaceae including 81 that are endemic, and many are threatened by both habitat loss and herbivory by other organisms. Initially a program was implemented to slow its spread, then an eradication attempt commenced in November 2012. The P. brassicae population was distributed over an area of approximately 100 km2 primarily in urban residential gardens. The eradication attempt involved promoting public engagement and reports of sightings, including offering a bounty for a two week period, systematically searching gardens for P. brassicae and its host plants, removing host plants, spraying insecticide to kill eggs and larvae, searching for pupae, capturing adults with nets, and augmenting natural enemy populations. The attempt was supported by research that helped to progressively refine the eradication strategy and evaluate its performance. The last New Zealand detection of P. brassicae occurred on 16 December 2014, the eradication program ceased on 4 June 2016 and P. brassicae was officially declared eradicated from New Zealand on 22 November 2016, 6.5 years after it was first detected and 4 years after the eradication attempt commenced. This is the first species of butterfly ever to have been eradicated.
**Order of Authors:**	Craig B. Phillips, PhD
	Kerry Brown
	Chris Green
	Richard Toft
	Graham Walker
	Keith Broome
**Additional Information:**	
**Question**	**Response**
**Financial Disclosure**<br><br>Enter a financial disclosure statement that describes the sources of funding for the work included in this submission. Review the submission guidelines for detailed requirements. View published research	Details of all costs are publicly available on-line in the Department of Conservation's 2015-16 Pieris brassicae eradication program annual report: www.doc.govt.nz/about-us/science-publications/conservation-publications/threats-and-impacts/animal-pests/pieris-brassicae-great-white-butterfly-eradication-annual-report/<br>Operational aspects of the eradication program were funded by the New Zealand Department of Conservation (DOC; www.doc.govt.nz). Vegetables New Zealand (www.freshvegetables.co.nz) contributed some funds to DOC to support operational

Powered by Editorial Manager® and ProduXion Manager® from Aries Systems Corporation

articles from *PLOS ONE* for specific examples.<br><br>This statement is required for submission and **will appear in the published article** if the submission is accepted. Please make sure it is accurate.<br><br>**Unfunded studies**<br>Enter: *The author(s) received no specific funding for this work.*<br><br>**Funded studies**<br>Enter a statement with the following details:<br>• Initials of the authors who received each award<br>• Grant numbers awarded to each author<br>• The full name of each funder<br>• URL of each funder website<br>• Did the sponsors or funders play any role in the study design, data collection and analysis, decision to publish, or preparation of the manuscript?<br>• **NO** - Include this sentence at the end of your statement: *The funders had no role in study design, data collection and analysis, decision to publish, or preparation of the manuscript.*<br>• **YES** - Specify the role(s) played.<br><br>* typeset	aspects of the eradication program.<br>KB CG, and KB are full time employees of DOC, which fully supported their research contributions. DOC also partly supported the research contributions of RT, who is a private consulting entomologist (www.entecol.co.nz).<br>The New Zealand government research institutes AgResearch (www.agresearch.co.nz) and Plant and Food Research (www.plantandfood.co.nz) are partners in a New Zealand research collaboration called Better Border Biosecurity (www.b3nz.org). The collaboration aims to help reduce the rate at which non-native insects, weeds and diseases that could harm valued New Zealand plants are becoming established in New Zealand. CP is an employee of AgResearch and GW of Plant and Food Research, and their research contributions to the eradication programme were funded largely via their employers' contributions to the Better Border Biosecurity collaboration.<br>The New Zealand Ministry for Primary Industries (www.mpi.govt.nz) also provided financial support for some of the research costs of CP, GW and RT, and the New Zealand TR Ellet Agricultural Trust contributed support for some of the research costs of CP.
**Competing Interests**<br><br>Use the instructions below to enter a competing interest statement for this submission. On behalf of all authors, disclose any competing interests that could be perceived to bias this work—acknowledging all financial support and any other relevant financial or non-financial competing interests.<br><br>This statement **will appear in the published article** if the submission is accepted. Please make sure it is accurate. View published research articles from *PLOS ONE* for specific examples.	The authors have declared that no competing interests exist.

Powered by Editorial Manager® and ProduXion Manager® from Aries Systems Corporation

**NO authors have competing interests**<br><br>Enter: *The authors have declared that no competing interests exist.*<br><br>**Authors with competing interests**<br><br>Enter competing interest details beginning with this statement:<br><br>*I have read the journal's policy and the authors of this manuscript have the following competing interests: [insert competing interests here]*<br><br>* typeset	
**Ethics Statement**<br><br>Enter an ethics statement for this submission. This statement is required if the study involved:<br><br>• Human participants<br>• Human specimens or tissue<br>• Vertebrate animals or cephalopods<br>• Vertebrate embryos or tissues<br>• Field research<br><br>Write "N/A" if the submission does not require an ethics statement.<br><br>General guidance is provided below. Consult the submission guidelines for detailed instructions. **Make sure that all information entered here is included in the Methods section of the manuscript.**	All work described in this manuscript that involved human subjects was conducted with strict adherance to legislation described in the New Zealand Biosecurity Act 1993 (http://www.legislation.govt.nz/act/public/1993/0095/latest/DLM314623.html). The data were collected by staff from the New Zealand Department of Conservation and Ministry for Primary Industries who were authorised to do so under the New Zealand Biosecurity Act 1993. Pieris brassicae is legislated as an unwanted organism under this Act, which means authorised persons have a wide range of statutory powers to enable them to control it; including accessing, inspecting and applying treatments on privately owned properties.

**Format for specific study types**<br><br>**Human Subject Research (involving human participants and/or tissue)**<br>• Give the name of the institutional review board or ethics committee that approved the study<br>• Include the approval number and/or a statement indicating approval of this research<br>• Indicate the form of consent obtained (written/oral) or the reason that consent was not obtained (e.g. the data were analyzed anonymously)<br><br>**Animal Research (involving vertebrate animals, embryos or tissues)**<br>• Provide the name of the Institutional Animal Care and Use Committee (IACUC) or other relevant ethics board that reviewed the study protocol, and indicate whether they approved this research or granted a formal waiver of ethical approval<br>• Include an approval number if one was obtained<br>• If the study involved *non-human primates*, add *additional details* about animal welfare and steps taken to ameliorate suffering<br>• If anesthesia, euthanasia, or any kind of animal sacrifice is part of the study, include briefly which substances and/or methods were applied<br><br>**Field Research**<br><br>Include the following details if this study involves the collection of plant, animal, or other materials from a natural setting:<br>• Field permit number<br>• Name of the institution or relevant body that granted permission	
**Data Availability**<br><br>Authors are required to make all data underlying the findings described fully available, without restriction, and from the time of publication. PLOS allows rare exceptions to address legal and ethical concerns. See the PLOS Data Policy and FAQ for detailed information.	No - some restrictions will apply

Powered by Editorial Manager® and ProduXion Manager® from Aries Systems Corporation

A Data Availability Statement describing where the data can be found is required at submission. Your answers to this question constitute the Data Availability Statement and **will be published in the article**, if accepted.<br><br>**Important:** Stating 'data available on request from the author' is not sufficient. If your data are only available upon request, select 'No' for the first question and explain your exceptional situation in the text box.<br><br>Do the authors confirm that all data underlying the findings described in their manuscript are fully available without restriction?	
**Describe where the data may be found in full sentences. If you are copying our sample text, replace any instances of XXX with the appropriate details.**<br><br>• If the data are **held or will be held in a public repository**, include URLs, accession numbers or DOIs. If this information will only be available after acceptance, indicate this by ticking the box below. For example: *All XXX files are available from the XXX database (accession number(s) XXX, XXX.).*<br>• If the data are all contained **within the manuscript and/or Supporting Information files**, enter the following: *All relevant data are within the manuscript and its Supporting Information files.*<br>• If neither of these applies but you are able to provide **details of access elsewhere**, with or without limitations, please do so. For example:<br><br>*Data cannot be shared publicly because of [XXX]. Data are available from the XXX Institutional Data Access / Ethics Committee (contact via XXX) for researchers who meet the criteria for access to confidential data.*<br><br>*The data underlying the results presented in the study are available from (include the name of the third party*	All relevant data are within the manuscript and its Supporting Information files.

and contact information or URL).<br>• This text is appropriate if the data are owned by a third party and authors do not have permission to share the data.<br><br>* typeset	
Additional data availability information:	Tick here if your circumstances are not covered by the questions above and you need the journal’s help to make your data available.

Powered by Editorial Manager® and ProduXion Manager® from Aries Systems Corporation

Manuscript

Click here to access/download;Manuscript;large_white_butterfly_eradication

Eradicating large white butterfly from New Zealand eliminates a threat to endemic Brassicaceae

Craig B. Phillips [a,b,*], Kerry Brown [c], Chris Green [c], Richard Toft [d], Graham Walker [b,e], Keith Broome [c]

[a] AgResearch, Private Bag 4749, Christchurch 8140, New Zealand

[b] Better Border Biosecurity research collaboration, www.b3nz.org

[c] Department of Conservation, PO Box 10-420, Wellington 6143, New Zealand

[d] Entecol Ltd, PO Box 2256, Stoke, Nelson 7041, New Zealand

[e] Plant & Food Research, Private Bag 92169, Auckland 1142, New Zealand

* Corresponding author at: AgResearch, Private Bag 4749, Christchurch 8140, New Zealand. Email address: craig.phillips@agresearch.co.nz

Abstract

In May 2010 the large white butterfly, *Pieris brassicae* L. (Lepidoptera: Pieridae), was discovered to have established in New Zealand. It is a Palearctic species that—due to its wide host plant range within the Brassicaceae—was regarded as a risk to New Zealand's native brassicas. New Zealand has 86 native species of Brassicaceae including 81 that are endemic, and many are threatened by both habitat loss and herbivory by other organisms. Initially a program was implemented to slow its spread, then an eradication attempt commenced in November 2012. The *P. brassicae* population was distributed over an area of approximately 100 km^2 primarily in urban residential gardens. The eradication attempt involved promoting public engagement and reports of sightings, including offering a bounty for a two week period, systematically searching gardens for *P. brassicae* and its host plants, removing host plants, spraying insecticide to kill eggs and larvae, searching for pupae, capturing adults with nets, and augmenting natural enemy populations. The attempt was supported by research that helped to progressively refine the eradication strategy and evaluate its performance. The last New Zealand detection of *P. brassicae* occurred on 16 December 2014, the eradication program ceased on 4 June 2016 and *P. brassicae* was officially declared eradicated from New Zealand on 22 November 2016, 6.5 years after it was first detected and 4 years after the eradication attempt commenced. This is the first species of butterfly ever to have been eradicated.

Keywords: invasive; non-native; alien; pest; impact; endemic plant; threat; urban; public awareness; sightings

1. Introduction

Unintentional introductions of nonnative species, including arthropods, are contributing to declining global biodiversity (Cicconardi et al., 2017; Vitousek et al., 1997; Wardle et al., 2011). Eradicating destructive nonnative species is challenging, but when successful can provide substantial benefits (Jones et al., 2016; Myers et al., 1998). The first organised attempt to eradicate a nonnative arthropod probably began in 1890 against the gypsy moth, *Lymantria dispar,* in the USA (Liebhold et al., 2016). Subsequently over 1200 programs in about 100 countries have attempted to eradicate at least 138 insect species (Kean et al., 2019). About 285 attempts (24%) have targeted 27 Lepidoptera species, which have all been moths rather than butterflies (Kean et al., 2019).

In May 2010, the Palaearctic large white butterfly, *Pieris brassicae* L. (Lepidoptera: Pieridae), was detected for the first time in New Zealand in Nelson (Fig. 1; Richardson and Voice, 2010). It had previously been accidentally introduced to South Africa (Geertsema, 1996) and Chile (Gardiner, 1974), and may have reached Nelson via its seaport as pupae on imported shipping containers, which is a known pathway for *P. brassicae* (Anonymous, 2002; Molet, 2011).

All of *P. brassicae*'s many host plants are brassicas (Brassicaceae) (Feltwell, 1982). Each female lays about 500 eggs, which are laid on host plants in batches of 50–150 eggs (Gardiner, 1963). Larvae feed gregariously and may defoliate several plants during their development. Fifth instar larvae crawl away from their host plants to pupate, typically on vertical surfaces in sheltered locations (Feltwell, 1982).

New Zealand has 86 native brassica species, of which 81 are endemic. Fifty seven have received threat classifications under a New Zealand system that was adapted from the International Union for Conservation of Nature Red List (Townsend et al., 2008): Twenty seven New Zealand brassicas are listed as Nationally Critical; eight are Nationally Endangered; six are Nationally Vulnerable; two are Declining; and 12 are Naturally Uncommon (de Lange et al., 2018). A further two species are presumed extinct, and ten are presumed threatened but are too data deficient to rank (S. Courtney, DOC, pers. comm. 2018). Many occur in small isolated populations that are expensive to protect and vulnerable to various threats, including herbivory by the closely related butterfly *P. rapae*; this species was accidentally introduced to New Zealand in 1930 (Hasenbank et al., 2011). *Pieris brassicae* also posed a risk to cultivated brassicas in New Zealand.

The Ministry for Primary Industries (MPI) is New Zealand's lead biosecurity agency with responsibilities to protect New Zealand's environment, economy, health and socio-cultural values under the Biosecurity Act 1993. MPI responded to *P. brassicae* by alerting the public, establishing a monitoring program to slow its spread and evaluating an eradication attempt. *Pieris brassicae* adults migrate long distances in Europe (Spieth and Cordes, 2012), which suggested it could spread quickly in New Zealand, and this impression was reinforced by *P. rapae* which took just 5–8 years to spread throughout New Zealand (Muggeridge, 1942). Surprisingly, however, *P. brassicae* still appeared to be restricted to Nelson 2 years after it was first recorded there (Philip, 2012). Nevertheless, MPI terminated its response in November 2012 because it considered an eradication attempt would probably fail and the expected benefit to cost ratio was too small (Brown et al., 2019).

New Zealand's Department of Conservation (DOC) is responsible for protecting native biodiversity under the Conservation Act 1987 and was concerned that the cold tolerance and dispersal ability of *P. brassicae* would put all New Zealand endemic brassica populations at risk except those on sub Antarctic islands (Phillips and Kean, 2013). Indeed, some vulnerable populations were within 10 km of Nelson. Accordingly, in November 2012 DOC began the first-ever attempt globally to eradicate a butterfly.

The operational details of many previous eradication programs reside in relatively inaccessible grey literature, which limits opportunities for learning (Genovesi, 2005; Simberloff, 2009, 2002). This paper aims to help inform future eradication programs by summarising the methods used and results obtained.

2. Methods

We define a 'detection' as the discovery of one or more *P. brassicae* at one location at one time. Thus, detections refer to the number of inspections that revealed *P. brassicae* rather than to the number of *P. brassicae* individuals found.

2.1 Management and review

A strategy was prepared before the eradication attempt commenced that documented the program's goal, objectives, actions, timeframes, stopping rules, and staff roles and responsibilities (Toft et al., 2012). A Coordinated Incident Management System (CIMS) framework was used to structure roles and

responsibilities (New Zealand Government, 2014; Additional Information 1). The program implemented a cycle of “plan, implement, monitor, report and review”, and emphasised team work, effective communication, and openness to suggestions for improvement.

A Technical Advisory Group (TAG) of six people with expertise in eradication and invertebrate ecology was assembled and led by DOC (author K. Brown). It produced plans; provided advice on the scale, intensity and timing of the response; conducted research; lobbied for financial support; and reported results. The group comprised three animal pest technical advisors from DOC including an entomologist, two entomologists from two government research institutes, and a private consulting entomologist.

The TAG assessed program feasibility in November 2013 (Phillips et al., 2013b), and the program was reviewed in August 2013 and December 2013. The first review was conducted by DOC and sought to both confirm the program was being well managed and identify opportunities for improvement (Briden and Broome, 2013). The second review was conducted by MPI and had similar goals to the first, plus it also evaluated the program’s likelihood of success (Gill, 2013a). Participants included three TAG members, nine independent experts, and five MPI staff (Gill, 2013b). Prior to the review, participants were sent a report describing program progress (Phillips et al., 2013a).

From 2013 to 2015, DOC managers were provided with estimates of the probability of eradication success. Five TAG members and another expert independently provided estimates using nine criteria developed by the TAG (Phillips et al., 2019) and the range and mean were reported to managers. Progress was also publicly reported via a series of annual reports (Phillips et al., 2014a, 2015a, 2016; Toft, 2013).

2.2 Operational area

An area of ca. 10000 ha was intensively managed during the eradication attempt and is termed the ‘operational area’. It included Nelson City (41.29°S, 173.28°E), the adjoining urban area of Richmond, and farmland (Fig. 1). It was populated by ca. 47000 people living in ca. 28000 households, and the main *P. brassicae* host plants present were brassica vegetables in home gardens, and nasturtium (*Tropaleum majus*) in gardens and wasteland. Some naturalised brassicas were also present

(Phillips et al., 2013a). Commercial brassica crops mainly occurred outside the operational area.

Nelson has a temperate oceanic climate with a summer average maximum temperature of 22 °C and a summer minimum of 12 °C. Winter average maximum and minimum temperatures are 14 °C and 4 °C. Average annual rainfall is 1043 mm, and average annual sunshine is 2449 hours. Mountains border Nelson's eastern perimeter from the south to the northeast, ocean lies to the northwest, and to the southwest is an intensively farmed plain.

To facilitate management, the operational area was divided into 46 management blocks (Additional Information 2) with areas ranging from 27–1944 ha. Within blocks, the units searched were mostly residential properties, though some commercial properties and public green spaces were also searched. Properties per block ranged from just two in a block that was predominantly farm land to ca. 2000.

2.3 Active surveillance

We define active surveillance as planned systematic searching for *P. brassicae* by DOC staff.

2.3.1 Field staff

All field staff underwent police vetting and employment checks prior to appointment and received Authorised Persons training to give them legal access to private properties without landowner permission under the New Zealand Biosecurity Act 1993. Training included communicating with property owners, managing aggressive dogs, first aid, identifying *P. brassicae* and its host plants (Anonymous, 2013), search methods, handling and applying pesticides, and data recording.

The eradication attempt began in November 2012 with only three field staff. As the scale of the eradication challenge became clearer, this number was increased to 24 by April 2013 and to 35 by November 2014. Field staff were divided into eight teams, each comprising 2–8 people. Six teams searched for *P. brassicae*, one specialised in controlling larger areas of host plants, and one responded to residents' reports of sightings and reinspected previously treated properties. Teams were issued with VHF and UHF radios, and team leaders carried mobile phones. Each day teams were assigned to search particular properties specified via analysis of previous surveillance results (see below).

2.3.2 Prioritising locations to search

The program aimed first to eliminate *P. brassicae*, then to continue surveillance to confirm eradication. During the elimination phase, the program prioritised the destruction of small peripheral *P. brassicae* populations to minimise spread beyond the operational area, while simultaneously treating the larger central population to reduce population growth and emigration pressure (Brown et al., 2013). During spring and autumn, the emphasis was on properties with host plants in blocks exhibiting comparatively high detection rates. Search locations were regularly reprioritised based on recent surveillance results, plus factors such as logistics and season (Phillips, 2014). During elimination, locations where *P. brassicae* and its host plants had seldom been recorded were searched relatively infrequently and mostly in summer or winter.

The program's transition from elimination to monitoring demanded confidence that *P. brassicae* was absent from the entire operational area, including locations infrequently searched during the elimination phase. Again, the emphasis of searching was on properties with host plants. Allocating search effort across all 46 blocks to maximise confidence *P. brassicae* had been eradicated was informed by a model that estimated relative probabilities of *P. brassicae* being present in each block (Kean and Phillips, in preparation; Phillips et al., 2016).

2.3.3 Search timing and frequency

The phenology of *P. brassicae* was modeled (Kean and Phillips, 2013, in preparation) using published data for its developmental responses to temperature (e.g., Davies and Gilbert, 1985) and day length (e.g., Spieth and Sauer, 1991). The model was validated against observations of *P. brassicae* both in the Northern Hemisphere and New Zealand, and helped to define the timing and frequency of searches.

Pieris brassicae had 2–4 generations per year in Nelson. Most *P. brassicae* overwintered as pupae, from which adults emerged in spring to lay eggs. In summer, approximately half of the population aestivated as pupae, with second generation adults emerging in autumn, which coincided with the emergence of third and fourth generation adults emerging from non-aestivating pupae (Kean and Phillips, 2013).

Pieris brassicae pupae were difficult to find (Phillips et al., 2014b) and prevailed in summer and winter. Thus, during these seasons all blocks were surveilled for host plants to enable the highest risk properties to be targeted the following autumn or spring when other more detectable life stages predominated. Nevertheless, some searching for pupae was also conducted in winter (see below).

During spring and autumn, consecutive bouts of surveillance in the same location occurred at different intervals depending on if and when *P. brassicae* had been detected there. In general, the program aimed to search properties in high priority blocks frequently enough to prevent any *P. brassicae* eggs laid after the previous search from becoming pupae before the next search; ca. every 2–4 weeks. However, if *P. brassicae* was detected on a property, the property was searched again before any eggs overlooked in the previous search could reach the pupal stage; ca. every 1–2 weeks. Reinspections of infested properties usually continued until no *P. brassicae* had been detected in two consecutive inspections. These short interval reinspections enabled the efficacy of searches for *P. brassicae* to be estimated (Phillips et al., 2014b).

2.3.4 Search methods

Properties were visited during the day and, if residents were present, permission to search was requested. If residents were absent, gardens were searched for *P. brassicae* and its host plants, and notification of the search was left. When properties could not be searched (e.g., due to threatening dogs, locked gates or unhelpful residents), contact was made again by phone or letter and access arranged.

Eggs and larvae were sought by systematically inspecting all host plants. Any found were removed, then host plants were treated. Immature *P. brassicae* were either killed upon detection, or kept in captivity to monitor parasitism then killed.

Pupae were searched for throughout the year, but were explicitly targeted during winter on properties where mid–late stage larvae had been detected the previous autumn. Inanimate objects such as fences, garden sheds and house exteriors were searched using ladders and torches as necessary to inspect cracks and crevices. Adjacent properties were also searched if it was suspected that larvae had crawled off the property to pupate.

Adults were searched for in sunny locations with abundant nectar sources and captured with hand-held nets. This was often difficult and time consuming due to *P. brassicae*'s rapid and evasive flight, but was considered worthwhile because: Capturing gravid females minimised the number of eggs they could otherwise have laid, potentially over many hectares; and capturing males when adult populations were low potentially inhibited mate finding and reduced female fecundity.

Research was conducted to develop attractants for *P. brassicae* adults, but did not produce practicably useful results (Sullivan et al., 2014; GP Walker et al., 2013). However in 2014 a DOC staff member, W. Wragg, developed an ultra-violet (UV) reflective lure that was attractive to *P. brassicae* adults. Its efficacy was optimised by measuring the UV reflectivity of various materials (Phillips et al., 2015b) to identify one with similar reflectivity to *P. brassicae* wings (e.g., Obara et al., 2008; Stavenga and Arikawa, 2006). A cloth with suitable UV reflectivity was glued to ornamental butterflies' wings, which moved by solar power, and the models were used to attract *P. brassicae* adults towards staff with nets.

2.4 Passive surveillance

Publicity aimed to engender support for the eradication program and promote reports of *P. brassicae*, and occurred at times when *P. brassicae* adults, eggs and larvae were about to appear. Communication methods included: DOC's website; a Facebook page; newspapers; magazines; billboards; leaflets and letters dropped in letter boxes; information displays and fridge magnet giveaways at events; face to face discussions with vegetable sellers and other groups; public talks; school visits; thank you cards to helpful property owners; newsletters regularly sent to stakeholders; advertisements at a local cinema; and advertisements, interviews and articles on local and national radio stations. Information given included descriptions of risks associated with: Accidentally moving *P. brassicae* pupae out of Nelson on vehicles such as campers and caravans, which are often stored near gardens; accidentally moving *P. brassicae* larvae out of Nelson on home-grown brassica seedlings, vegetables and vegetable waste; and use of brassicas as winter cover crops. Automobile mechanics were asked to be vigilant for *P. brassicae* pupae when conducting safety checks of vehicles, trailers, and caravans. Interpreters were employed to talk to recent New Zealand immigrants in their first language. The public were asked to report sightings of *P. brassicae* via a continuously monitored toll-free

number operated by MPI. Reports were immediately conveyed to DOC, which responded within 48 hours, usually visiting the properties for verification.

2.4.1 Bounty hunt

A NZ$10 bounty was offered for each dead *P. brassicae* adult given to DOC during a 2 week school holiday in spring 2013. The bounty was only offered for this one period to minimise any motivation to culture *P. brassicae* for profit.

2.5 Population delimitation

Monitoring for *P. brassicae* outside the operational area occurred via active surveillance, passive surveillance, monitoring of native brassica populations by DOC, and searching commercial brassica crops by staff from a nearby crop research institute, who searched for *P. brassicae* when conducting routine scouting for other pests in brassica crops.

2.6 Treatments

2.6.1 Insecticides

A program review recommended that all *P. brassicae* host plants at a site should be sprayed with insecticide whenever eggs or larvae were found because search efficacy was likely < 100% (Briden and Broome, 2013). Consequently, the BioGro-certified organic insecticide Entrust® SC Naturalyte® was chosen as the most socially acceptable option. The horticultural oil D-C-Tron® was added to improve spray coverage and increase egg mortality. Spraying was usually conducted after gaining consent from property occupants, but occasionally occurred without consent when the occupants could not be contacted and late-stage larvae were found. If occupants were opposed to this treatment then one of the following alternatives were used: Either removing or regularly inspecting host plants, or applying a microbial insecticide containing toxins from the bacterium *Bacillus thuringiensis* (Bt).

2.6.2 Host plant control

Host plant patches were prioritised for control based on their size and proximity to *P. brassicae* detections, and treated sites were reinspected to verify treatment efficacy. Staff with abseiling experience accessed host plants on steep terrain. Nasturtium

growing in unpopulated areas was treated with a mixture of glyphosate, a desiccant (carfentrazone-ethyl), a surfactant, plus an insecticide in case any *P. brassicae* were present.

2.6.3 Biological control

During the 1930s, two parasitic wasp species were introduced to New Zealand for biological control of *P. rapae*: *Cotesia glomerata* L. (Hymenoptera: Braconidae), which parasitises larvae, and *Pteromalus puparum* L. (Hymenoptera: Pteromalidae), which parasitises late-stage larvae and pupae (Muggeridge, 1943). Both species also parasitise *P. brassicae* (Muggeridge, 1943) and were present in Nelson before *P. brassicae* was detected there.

Parasitism of *P. brassicae* by *C. glomerata* within the operational area was evaluated from October 2013 until June 2014 during active surveillance. *Pieris brassicae* larvae were subsampled (ca. 10 larvae per brood) and individuals were placed in separate pottles with brassica leaf for food then reared to fate (adulthood, death or parasitoid emergence) (Walker et al., 2014).

To attempt to augment parasitism in the operational area, *C. glomerata* cocoons were collected from *P. rapae* infestations in several New Zealand locations (G Walker et al., 2013; Walker et al., 2014) and from *P. brassicae* infestations in Nelson. Cocoons were maintained until adult emergence, and adults were provided with sugar solution and allowed to mate. During autumn 2014 and autumn 2015, *C. glomerata* adults were released in locations where there had been either: Recent repeated *P. brassicae* detections; recent detections in areas that were difficult to search; or few recent searches. No attempt was made to evaluate if the releases increased parasitism rates.

In autumn 2015, laboratory cultured *Pt. puparum* were released as larvae developing within *P. rapae* pupae at locations where there was a high risk of *P. brassicae* late-stage larvae and pupae being present (Richards et al., 2016). To measure if the releases increased parasitism rates, unparasitized sentinel *P. rapae* pupae were situated in cages accessible to *Pt. puparum* adults either within 2–3 m of the release locations, or > 200 m from them, then monitored for parasitism (Richards et al., 2016).

2.7 Data collection and management

Data management was continuously refined and ultimately rested on a Geospatial Information System (GIS) built on an Environmental Services Research Institute ArcGIS Server. Web GIS (Geocortex Essentials) Version 4.4.2 was used to enter property inspection data. ArcGIS Version 10.3.1 was used to analyse spatial data and produce interactive maps, with dynamic queries indicating the highest priority properties to surveil. It was also used to help update the underlying Nelson cadastre to ensure that teams visited the correct addresses.

Field teams took a map of locations to be searched, conducted the inspections, and manually recorded details of any *P. brassicae*, host plants and access issues (Additional Information 3). This information was transferred to the GIS typically within 48 hours and used to produce updated maps for subsequent surveillance. A data analyst refined processes for data entry, capture, storage and analysis, and developed models that provided staff with access to reports on factors such as blocked access, safety (e.g. aggressive dogs), surveillance results, host plant control, and properties to be searched.

2.8 Preparing this paper

Data were manipulated and Figures 1–3 created using the statistical programming language R version 3.6.0 (R Core Team, 2019) and functions in the R packages 'tidyverse' (Wickham, 2017), 'sf' (Pebesma, 2018) and 'ggsn' (Baquero, 2019). Figures 1 and 3 used data sourced from the Land Information New Zealand Data Service licensed for reuse under CC BY 4.0.

3. Results

3.1 Management and review

The September 2013 feasibility assessment (Phillips et al., 2013b) concluded that seven of the nine criteria of Phillips et al. (2019) were being substantially met whereas two were only being marginally met: These were (i) *Irrespective of its density, the population can be forced to decline from one year to the next,* and (ii) *Immigration and emigration can be prevented*.

DOC's August 2013 review made recommendations, all subsequently implemented, to increase insecticide use on infested properties, prepare a formal communication plan, and increase public awareness and community involvement in the program (Briden and Broome, 2013). MPI's December 2013 review concluded

that the program was being appropriately managed, it was too early to evaluate feasibility, and the program was worth continuing, but was concerned about *P. brassicae* escaping from the operational area (Curran, 2013).

An October 2013 estimate of the program's probability of success had a mean of 56% (range 50–60 %, n = 6). However, the estimates increased in November 2014 to 80 % (range 70–92 %, n = 6) and in July 2015 to 91 % (range 81–98 %, n = 6).

3.2 Active surveillance

Repeated inspections of infested properties enabled the efficacy of searches for *P. brassicae* to be estimated (Phillips et al., 2014b). Following a single inspection, the proportion of properties where eggs or larvae were detected during the subsequent inspection declined from 32–52% in April–May 2013 when most staff were inexperienced to 5–25% in September–October 2013 when staff were fully trained. After late 2013 when insecticide use on infested properties increased, the proportion of properties where some *P. brassicae* eggs or larvae remained after an inspection declined to 1–11%. Thus, an insecticide treatment plus just one follow up inspection were sufficient to ensure all eggs and larvae had been eliminated from ≥ 99 % of infested properties (Phillips et al., 2014b). However, the program generally maintained two follow up inspections to maximise treatment efficacy.

Early in the program, field staff suspected that infested properties occurred in clusters with radii of ca. 50–250 m. Thus, when *P. brassicae* was detected on a property, an early practice was to also inspect adjacent properties within these radii (Phillips et al., 2014a). However, a spatial analysis of surveillance data found no evidence for clustered detections, thus it was concluded that searching properties that surround an infested property was unlikely to increase detection rates above searching randomly chosen properties in the same block (Phillips and van Koten, 2014) and the practice was discontinued. Further evidence that individual *P. brassicae* females often oviposited in disparate locations 2–5 km apart was obtained by analysing genetic variation in the mitochondrial COI gene of all detected specimens (Hiszczynska-Sawicka and Phillips, 2014). Because the location and life stage of every detected specimen had been recorded, the spatial distributions of potential offspring of each captured female could be modelled by matching the

mitochondrial genotypes of female and immature *P. brassicae* while assuming a range of values for female longevity (Phillips, Sawicka and Kean, unpublished).

The UV lures were first deployed in October 2014 when detection rates had already declined to low levels (Fig. 2). *Pieris brassicae* adults approached the lures in a manner similar to *P. rapae* (Obara et al., 2008a, b), but never alighted on them. From 10 October 2014 to 3 November 2014, it took 180 person-hours to capture three *P. brassicae* adults without a lure, whereas it took 44 person-hours to capture seven with a lure.

Overall, field staff conducted ca. 260000 inspections, of which ca. 3000 (1 %) detected *P. brassicae* (Phillips et al., 2016). At any one time, ca. 60% of residential properties had gardens and ca. 40% had *P. brassicae* host plants, though the actual properties making up these proportions varied with time, thus necessitating ongoing monitoring to track properties with host plants. The most abundant host plant in Nelson was nasturtium and ca. 35% of detections occurred on this plant (Phillips et al., 2014a). A similar proportion of detections occurred on broccoli, even though it was recorded less frequently in Nelson, which suggested it was a preferred host (Phillips et al., 2014a).

3.3 Passive surveillance

A bounty for *P. brassicae* was offered for 2 weeks in spring 2013. In all, 319 individuals or groups handed in 3268 adults comprising 133 *P. brassicae* (4 %) and 3135 *P. rapae* (96 %) (Phillips et al., 2013a). The *P. rapae* were from locations up to 130 km from Nelson, whereas *P. brassicae* only came from within the operational area.

The public submitted 1936 reports (additional to the bounty) of which 586 (30 %) proved to be *P. brassicae* (Phillips et al., 2016). Most reports (76 %) were made via the toll-free number, and the remainder were largely reported by phone directly to DOC's office in Nelson (Phillips et al., 2016).

3.4 Temporal changes in spatial distribution

Pieris brassicae was first detected in May 2010 and by October 2010 it had been found at eight properties in urban Nelson up to 12 km apart (Philip, 2010). Over the next 2 years, passive surveillance reports suggested its distribution had not dramatically changed (Philip, 2012) (Fig. 3, 'Before 1 Dec 2012').

When the eradication program began in summer 2012, there were several detections outside the operational area. In summer 2012-13 (Fig. 3), one (parasitised) *P. brassicae* larva was found ca. 25 km west of Port Nelson near Upper Moutere (Fig. 1). This required intensive work to gain confidence additional *P. brassicae* had not escaped from the operational area, including increased publicity between Upper Moutere, Motueka and Nelson (Fig. 1). The larva was likely taken to Upper Moutere from Nelson on an infested cabbage. Between autumn 2013 and autumn 2014 (Fig. 3), several *P. brassicae* were detected ca. 11 km north of Port Nelson at Glenduan (Fig. 1), which also required significant treatment. In summer 2013-14 (Fig. 3), one adult was detected ca.15 km southwest of Port Nelson at Hope and another was detected ca. 10 km northeast of Port Nelson at Lud Valley (Fig. 1). Intensive searching in the vicinities of these detections revealed no further *P. brassicae*.

Despite such dispersal events, from autumn 2014 *P. brassicae* became increasingly confined to central Nelson (Fig. 3), and it became apparent during 2016 that the last detection had occurred near central Nelson in summer 2014-15 (Fig. 3). Thereafter, active surveillance persisted until winter 2016 when confidence that *P. brassicae* had been eliminated was sufficient to terminate the program (Fig. 3).

3.5 Temporal changes in detection rates

Eggs, larvae and adults of *P. brassicae* were more detectable than pupae, thus there were peaks in detection rates during spring and autumn when they were more prevalent than pupae (Fig. 2). Monthly rates peaked in September 2013 when *P. brassicae* (including all life stages) was detected on 9% of 2931 inspected properties. By this time, staff had been fully trained, *P. brassicae* was relatively abundant, and most of the population was exposed to control (i.e., few pupae). Thereafter, rates generally declined, though they showed regular smaller peaks each autumn and spring until the end of 2014. They declined to zero in January 2015 and remained there until 4 June 2016 when surveillance ended (Fig. 2).

3.6. Treatments

3.6.1 Insecticides

Following a detection, ca. 30 % of property owners asked for an alternative treatment to Entrust® SC Naturalyte®: About 20 % chose host plant removal, 5 % chose regular host plant checks, and the remainder chose Bt (Phillips et al., 2015a).

3.6.2 Host plant control

Host plants were controlled on a mean of 2620 ± 489 (± SD) properties per year, with some properties treated up to three times annually to manage regrowth. Specialist abseiling skills and/or commercial herbicide sprayers were needed to apply treatments on ca. 15 properties per year. Nasturtium and other naturalised brassicas such as wallflower (*Erysimum* spp.) most often required specialist attention, with patches of up to 500 m^2 present in some steep locations.

3.6.3 Biological control

Monitoring of *C. glomerata* parasitism of *P. brassicae* during October 2013–June 2014 revealed that 65% of *P. brassicae* broods (n = 130) contained *C. glomerata*, and a mean of 35% of larvae (n = 999) per brood were parasitised (Walker et al., 2014). To augment parasitism, ca. 10000 *C. glomerata* adults were released in the operational area during autumn 2014 and a further ca. 6600 were released in autumn 2015, though it is unknown if this increased parasitism rates (Phillips et al., 2015a).

During autumn 2015, over 14000 *Pt. puparum* adults were released at 17 Nelson properties (Richards et al., 2016). Parasitism of sentinel *P. rapae* was rare—as were detections of *P. brassicae* pupae—and no effect of the releases on parasitism rates by *Pt. puparum* was detected (Richards et al., 2016).

3.7 Data collection and management

Early data entry issues included a GIS interface that: Allowed users to inadvertently enter incorrect/invalid inspection dates and misspelled addresses; and provided users with inadequate confirmation that new records had been successfully entered and saved, which often provoked duplicate entries. These issues were compounded by the Nelson cadastre initially being incomplete and out of date, which sometimes created confusion for field staff about the spatial locations of addresses and resulted

in inspection records being assigned to incorrect addresses. These problems created a dataset that was time-consuming to correct before it could be reliably used for analysis. In November 2014, a data manager with GIS expertise was assigned full time to the eradication program, and remaining issues with the cadastre and GIS interface were resolved by early 2015.

4. Discussion

The attempt to eradicate *P. brassicae* was officially declared successful by MPI and DOC in November 2016 (Klein, 2016), thus becoming New Zealand's 69th successful arthropod eradication (Kean et al., 2019). However, unlike many other successful programs in New Zealand and elsewhere, powerful detection tools such as pheromone traps were unavailable for *P. brassicae*, and detection largely depended on host/habitat searches. A meta analysis of arthropod eradication attempts (Tobin et al., 2014) found that programs relying on such methods were unlikely to succeed, though this effect became non-significant when programs directed against just two species for which effective detection methods are available, *Lymantria dispar dispar* (n = 73 programs) and *Ceratitis capitata* (n = 56), were excluded from analysis. Limitations of the available *P. brassicae* detection methods may have been partly compensated by *P. brassicae* eggs, larvae and adults being relatively conspicuous, and eggs and larvae having a distinctive appearance among New Zealand insects. People are more likely to report distinctive looking insects, particularly if they are pests (Caley et al., 2019). Moreover, *P. brassicae* eggs and larvae occurred on low growing, readily accessible host plants, and larval feeding damage often became increasingly conspicuous as defoliation proceeded. New Zealand conservationists, particularly DOC, have also had many successes eradicating mammalian pests for which there are few powerful detection tools (Clout and Russell, 2006; Russell and Broome, 2016; Towns et al., 2018).

Tobin et al. (2014) found that the probability of eradication success declined, and total program cost grew, with increasing infestation size. The *P. brassicae* infestation in New Zealand had a maximum extent of about 100 km^2 and previous attempts to eradicate similar sized infestations had a probability of success of about 0.75 (Tobin et al., 2014). The *P. brassicae* program cost US$3.28 million (NZ$4.97 million, €2.93 million), which was less than the approximately US$5 million predicted by the meta analysis (Tobin et al., 2014).

Numerous aspects of the eradication program additional to *P. brassicae*'s conspicuousness and accessibility likely contributed to its success at relatively low cost. The program engendered strong public support and received valuable reports of sightings that accounted for ca. 20% of all *P. brassicae* detections. This support was fostered by comprehensive publicity, rapid responses to public reports, respectful and communicative staff, and the availability of an effective organic insecticide which was more acceptable to many residents than synthetic chemical alternatives. The bounty particularly excited public interest, plus it eliminated some *P. brassicae* and provided independent evidence that the population had been correctly delimited. It was also helpful that in 2001 MPI had declared *P. brassicae* an Unwanted Organism under the New Zealand Biosecurity Act 1993 because it gave authorised staff the legal right to search and treat private properties for *P. brassicae*. Moreover, some DOC staff had this authorisation before the program began, and after it commenced they expedited training to authorise additional staff.

Sometimes when nonnative organisms are discovered in new regions, little technical information is available to support effective responses (Pluess et al., 2012). However, numerous studies of *P. brassicae* in its native range were available to support aspects of the eradication attempt including species diagnosis, identifying effective chemical treatments, defining the butterfly's host range and natural enemies, and developing a phenology model and lure. Unfortunately, such information had not been used to develop preparedness plans prior to the establishment of *P. brassicae* in New Zealand, which might have further increased the probability of eradication success (Pluess et al., 2012).

Several aspects of *P. brassicae*'s New Zealand habitat and ecology were fortuitously helpful to the program. Numerous *P. brassicae* natural enemies were present in Nelson and probably facilitated population suppression. These included: the insect parasitoids *C. glomeratus* and *Pt. puparum* (Muggeridge, 1943); and insect predators such as *Vespula vulgaris*, *V. germanica* (Brodmann et al., 2008), *Polistes chinenis antennalis* (Clapperton, 1999), various species of ants (Jones, 1987), spiders, harvestmen and predatory beetles (Dempster, 1967) and birds (Baker, 1970). The butterfly's potential population growth rate in Nelson was also limited by a proportion of the population entering aestivation, which reduced that part of the population's annual number of generations (Spieth et al., 2011; Kean and Phillips, 2013).

Throughout the program, doubt persisted that the feasibility criterion *Immigration and emigration can be prevented* (Phillips et al., 2019) could be met. The possibility that people would accidentally carry *P. brassicae* immatures beyond the operational area (e.g., on infested host material) and the ability of *P. brassicae* adults to fly long distances (Spieth and Cordes, 2012) meant there was constant potential for the pest to escape the operational area and establish elsewhere. This risk was partly mitigated by both comprehensive publicity and assiduous treatment of pest populations on the periphery of the operational area. Nelson's topography probably also helped to reduce emigration rates because ocean lies to its northwest, the mountains to its east contained few host plants, and arguably the sole benign pathway for natural dispersal was across the agricultural plains to its south. Moreover, the abundant and diverse *P. brassicae* natural enemies in New Zealand might have reduced the chance that emigrants could found new populations: Such biotic resistance has been observed in other insect host–natural enemy systems (Funderburk et al., 2016; Schulz et al., 2019).

An effective program structure, sound leadership, and emphases on assiduous field work, team spirit, open communication and an 'eradication attitude' (Brown and Brown, 2015) were undoubtedly curcial to the program's success, as was scientific support. However, some TAG members and scientists conducted work that was beyond their role if they possessed expertise that the program urgently needed. Examples included governance, project management, operational planning and management, data cleaning and analysis, and species diagnostics. Such role flexibility and commitment were important for maintaining the momentum of the eradication program whenever bottlenecks in staff numbers or expertise became evident.

The data management issues experienced predominantly during the first 2 years of the program reduced operational and analytical efficiency, but did not create serious doubt about achieving the feasibility criterion, "*Programme is effectively managed, and its status is reliably monitored and accurately recorded*" (Phillips et al., 2019). This was because it was always apparent that the data were being collected and corrected. However, the inefficiencies suffered would probably have been avoided by employing a qualified full-time data manager with access to a suitable GIS from the outset.

Although the eradication attempt was assisted by numerous factors, it still presented many ecological, technical and operational uncertainties (Brown et al., 2019) and, like most other eradication programs, it was complex (Vreysen et al., 2007; Simberloff et al., 2013). Quantifying benefits and assessing feasibility are important prerequisites to commencing an eradication program (Broome et al., 2005; Brown et al., 2019; Vreysen et al., 2007). With *P. brassicae*, an inability to measure the conservation values at risk in dollars terms and uncertainty about feasibility delayed the program's commencement by 2.5 years (Brown et al., 2019) even as *P. brassicae* population growth was increasing the eradication challenge. Nevertheless, the delay between detection and program commencement was less than the threshold of about 4 years beyond which eradication success becomes much less likely, as identified from a meta analysis of 173 eradication programs (Pluess et al., 2012).

The program began just as DOC was being restructured, which disrupted internal communication, created uncertainty about roles and budgets, and distracted managers. This culminated in the program receiving inadequate funding during January–June 2015 and being forced to reduce field staff, whose numbers were approximately halved during February–March 2015, then cut to zero during May–June 2015. However, in July 2015 the program's budget was renewed, many of the program's former field staff returned, and the eradication attempt recovered from what was widely perceived as a critical threat to its success. It subsequently became apparent that the last detection of *P. brassicae* had already occurred on 16 December 2014 and, critically, the renewed funding enabled the species' absence from Nelson to be demonstrated. The program ceased on 4 June 2016 and *P. brassicae* was officially declared eradicated from New Zealand on 22 November 2016 (Office of the Minister of Conservation, 2016), 6.5 years after it was first detected and 4 years after the eradication attempt commenced.

Acknowledgements

We thank the following DOC staff members for their patient and persistent efforts: Neil Clifton, who gave the go-ahead despite the uncertainty; Bruce Vander Lee and Mike Shephard, who were Project Managers; Simon Bayly and Julie Murphy, who were Operations Managers; James Reid, who built the GIS database; Jo Rees, who led planning; Senay Senait and Kath Henderson, who managed the data; Nicola

Gourlay, Eva Pomeroy and Rosemary Vander Lee, who managed logistics/ administration; Jaine Cronin, Sally Leggett and Trish Grant who made major contributions to community engagement; Dan Chisnall and Derek Brown, who managed host plant control; Keith Briden, who helped to review the program; and over 60 people who worked in the field. We also acknowledge the many people from MPI, AgResearch, Better Border Biosecurity (B3), Plant & Food Research and Vegetables New Zealand who generously provided their time, advice, support and expertise, particularly John Kean, Ela Sawicka and Nicky Richards (AgResearch/ B3). We also thank John Dugdale (Landcare Research) and Henk Geertsema (University of Stellenbosch) for their valuable advice, and the experts who reviewed the program in December 2013: Mandy Barron (Manaaki-Whenua Landcare Research), Jacqueline Beggs (University of Auckland), Ecki Brockerhoff (Scion), Stephen Goldson (AgResearch), Mark Hoddle (Center for Invasive Species Research, UC-Riverside, USA), Margaret Stanley (University of Auckland) and Patrick Tobin (USDA Forest Service, USA). Oluwashola Olaniyan (Lincoln University) provided helpful suggestions that improved the manuscript.

Role of funding sources

Operational aspects of the eradication program were funded by DOC and Vegetables New Zealand. Research to support the eradication program was funded by AgResearch, Better Border Biosecurity, the TR Ellet Agricultural Trust, MPI, and Plant & Food Research.

References

Anonymous, 2013. GWB Host Plant Guide, version 2. Department of Conservation.

Anonymous, 2002. NAPPO Phytosanitary Alert System [WWW Document]. URL https://www.pestalerts.org/viewArchNewsStory.cfm?nid=205 (accessed 7.10.19).

Baker, R., 1970. Bird predation as a selective pressure on the immature stages of the cabbage butterflies, *Pieris rapae* and *P. brassicae*. Journal of Zoology 162, 43–59.

Baquero, O. S., 2019. ggsn: North Symbols and Scale Bars for Maps Created with 'ggplot2' or 'ggmap'. R package version 0.5.0. https://CRAN.R-project.org/package=ggsn.

Briden, K., Broome, K., 2013. Great White Butterfly Review (No. DOCDM 1278224). Department of Conservation, Nelson.

Brodmann, J., Twele, R., Francke, W., Hölzler, G., Zhang, Q.-H., Ayasse, M., 2008. Orchids mimic green-leaf volatiles to attract prey-hunting wasps for pollination. Current Biology 18, 740–744.

Broome, K., Cromarty, P., Cox, A., 2005. Rat eradications-how to get it right without a recipe. Presented at the Proceedings of the 13th Australasian Vertebrate Pest Conference, Te Papa, Wellington, pp. 152–157.

Brown, K., Brown, D., 2015. Control to eradication of *Tradescantia fluminensison* Stephens Island (Takapourewa): the importance of systematic and persistent effort. DOC Research & Development Series 15.

Brown, K., Phillips, CB, Broome, K., Green, C., Toft, R, Walker, G., 2019. Feasibility of eradicating the large white butterfly (*Pieris brassicae*) from New Zealand: data gathering to inform decisions about the feasibility of eradication, in: Island Invasives: Scaling up to Meet the Challenge, Occasional Paper of the IUCN Species Survival Commission. International Union for the Conservation of Nature, Gland, Switzerland, pp. 364–369.

Brown, K., Toft, R., Van der Lee, B., 2013. Great White Butterfly Eradication Plan (No. DOCDM-1106615). DOC.

Caley, P., Welvaert, M., Barry, S.C., 2019. Crowd surveillance: estimating citizen science reporting probabilities for insects of biosecurity concern. Journal of Pest Science 1–8.

Cicconardi, F., Borges, P.A.V., Strasberg, D., Oromí, P., López, H., Pérez-Delgado, A.J., Casquet, J., Caujapé-Castells, J., Fernández-Palacios, J.M., Thébaud, C., Emerson, B.C., 2017. MtDNA metagenomics reveals large-scale invasion of belowground arthropod communities by introduced species. Mol Ecol n/a-n/a. https://doi.org/10.1111/mec.14037

Clapperton, B., 1999. Abundance of wasps and prey consumption of paper wasps (Hymenoptera, Vespidae: Polistinae) in Northland, New Zealand. New Zealand Journal of Ecology 23, 11–19.

Clout, M., Russell, J., 2006. The eradication of mammals from New Zealand islands. Assessment and Control of Biological Invasion Risks'.(Eds F. Koike, MN Clout, M. Kawamichi, M. De Poorter and K. Iwatsuki.) pp 127–141.

Curran, S., 2013. Minutes: Great White Butterfly [independent] Technical Advisory Group Meeting. Ministry for Primary Industries, Wellington.

Davies, C.R., Gilbert, N., 1985. A comparative study of the egg-laying behaviour and larval development of *Pieris rapae* L. and *P. brassicae* L. on the same host plants. Oecologia 67, 278–281. https://doi.org/10.1007/bf00384299

De Lange, P.J., Rolfe, J.R., Champion, P.D., Courtney, S., Heenan, P.B., Barkla, J.W., Cameron, E.K., Norton, D.A., Hitchmough, R., 2013. Conservation status of New Zealand indigenous vascular plants, 2012. Department of Conservation Wellington.

Dempster, J.P., 1967. The Control of *Pieris rapae* with DDT. I. The Natural Mortality of the Young Stages of *Pieris*. Journal of Applied Ecology 4, 485–500. https://doi.org/10.2307/2401350

Feltwell, J., 1982. Large white butterfly: the biology, biochemistry, and physiology of *Pieris brassicae* (Linnaeus). Springer.

Funderburk, J., Frantz, G., Mellinger, C., Tyler-Julian, K., Srivastava, M., 2016. Biotic resistance limits the invasiveness of the western flower thrips, *Frankliniella occidentalis* (Thysanoptera: Thripidae), in Florida. Insect science 23, 175–182.

Gardiner, B., 1974. *Pieris brassicae* (L.) established in Chile: another palearctic pest crosses the Atlantic (Pieridae). Journal of the Lepedopterists' Society 28, 269–277.

Gardiner, B., 1963. Genetic and environmental variation in *Pieris brassicae*. Journal of Research on the Lepidoptera 2, 127–136.

Geertsema, H., 1996. The large cabbage white, *Pieris brassicae*, an exotic butterfly of potential threat to cabbage growers in the Western Cape, South Africa. Journal of the Southern African Society of Horticultural Sciences 6, 31–34.

Genovesi, P., 2005. Eradications of invasive alien species in Europe: a review. Biological invasions 7, 127–133.

Gill, G., 2013a. Terms of Reference for the Great White Butterfly External Technical Advisory Group: *Pieris brassicae*. Ministry for Primary Industries, Wellington.

Gill, G., 2013b. Agenda: MPI & External Technical Advisory Group (Great White Butterfly) Technical Advisory Group Meeting. Ministry for Primary Industries, Wellington.

Hasenbank, M., Brandon, A., Hartley, S., 2011. White butterfly (*Pieris rapae*) and the white rust *Albugo candida* on Cook's scurvy grass (*Lepidium oleraceum*). New Zealand Journal of Ecology 35, 69–75.

Hiszczynska-Sawicka, E., Phillips, C., 2014. Mitochondrial cytochrome c oxidase subunit 1 sequence variation in New Zealand and overseas specimens of *Pieris brassicae* (Lepidoptera: Pieridae). New Zealand Plant Protection 67, 8–12.

Jones, H.P., Holmes, N.D., Butchart, S.H., Tershy, B.R., Kappes, P.J., Corkery, I., Aguirre-Muñoz, A., Armstrong, D.P., Bonnaud, E., Burbidge, A.A., 2016. Invasive mammal eradication on islands results in substantial conservation gains. Proceedings of the National Academy of Sciences 113, 4033–4038.

Jones, R.E., 1987. Ants, parasitoids, and the cabbage butterfly *Pieris rapae*. The Journal of Animal Ecology 56, 739–749.

Kean, J., Phillips, C., 2014. Optimal re-inspection intervals after detection of great white butterfly (*Pieris brassicae*) at Nelson properties (Report to Ministry for primary Industries No. RE400/2014/579). AgResearch, Lincoln.

Kean, J., Phillips, C., 2013. Phenology and diapause research for great white butterfly (*Pieris brassicae*). Milestone 4: Detailed spreadsheet model for phenology (Report for Ministry for Primary Industries No. RE400/2013/481), MPI 16341. AgResearch, Lincoln.

Kean, J., Suckling, D., Sullivan, N., Tobin, P., Stringer, L., Lee, D., Smith, G., Flores Vargas, R., Fletcher, J., Macbeth, F., 2019. Global eradication and response database [WWW Document]. Global eradication and response database. URL http://b3.net.nz/gerda/index.php (accessed 6.12.19).

Klein, A., 2016. New Zealand is the first country to wipe out invasive butterfly | newscientist.com [WWW Document]. New Scientist - Daily News. URL https://www.newscientist.com/article/2114573-new-zealand-is-the-first-country-to-wipe-out-invasive-butterfly/ (accessed 11.29.16).

Liebhold, A.M., Berec, L., Brockerhoff, E.G., Epanchin-Niell, R.S., Hastings, A., Herms, D.A., Kean, J.M., McCullough, D.G., Suckling, D.M., Tobin, P.C., 2016. Eradication of invading insect populations: from concepts to applications. Annual review of entomology 61, 335–352.

Molet, T., 2011. CPHST Pest Datasheet for *Pieris brassicae*. USDA-APHIS-PPQCPHST.

Muggeridge, J., 1943. The white butterfly (*Pieris rapae* L.) II. Parasites of the butterfly. New Zealand Journal of Science and Technology A 25, 1–18.

Muggeridge, J., 1942. The white butterfly (*Pieris rapae* L.) I. Its establishment, spread, and control in New Zealand. New Zealand Journal of Science and Technology A 24, 107–129.

Myers, J.H., Savoie, A., Randen, E. van, 1998. Eradication and pest management. Annual Review of Entomology 43, 471–491.

New Zealand Government, 2014. The New Zealand Coordinated Incident Management System (CIMS) (No. 2nd edition). Wellington.

Obara, Y., Koshitaka, H., Arikawa, K., 2008. Better mate in the shade: enhancement of male mating behaviour in the cabbage butterfly, *Pieris rapae crucivora*, in a UV-rich environment. Journal of Experimental Biology 211, 3698–3702.

Office of the Minister of Conservation, 2016. Great white butterfly eradication success: Media release 23 November 2016 [WWW Document]. URL http://www.doc.govt.nz/news/media-releases/2016/great-white-butterfly-eradication-success/ (accessed 11.24.16).

Pebesma, E., 2018. Simple Features for R: Standardized Support for Spatial Vector Data. The R Journal 10, 439-446, https://doi.org/10.32614/RJ-2018-009.

Philip, B., 2012. Biosecurity Response Business Case: Great white cabbage butterfly (No. Version 0.2 (final)). Ministry of Agriculture and Forestry, Biosecurity New Zealand, Wellington.

Philip, B., 2010. Consultation paper - *Pieris brassicae*, the great white cabbage butterfly, Investigation 2010-161. Biosecurity New Zealand, Wellington, New Zealand.

Phillips, C., 2014. Prioritising GWB management blocks (Report to MPI and DOC No. RE400 /2014 /607), AgResearch client report. AgResearch, Lincoln.

Phillips, C., Brown, K., Broome, K., Green, C., Walker, G., 2019. Criteria to help evaluate and guide attempts to eradicate arthropod pests, in: IUCN Island Invasives: Scaling up to Meet the Challenge., Occasional Paper of the IUCN Species Survival Commission. International Union for the Conservation of Nature, Gland, Switzerland, pp. 400–404.

Phillips, C., Brown, K., Green, C., Broome, K., Toft, R., Shepherd, M., Bayley, S., Rees, J., 2016. *Pieris brassicae* (great white butterfly) eradication annual report 2015/16 (Department of Conservation Annual Report). Department of Conservation, Nelson, New Zealand.

Phillips, C., Brown, K., Green, C., Broome, K., Toft, R., Shepherd, M., Bayley, S., Rees, J., 2015a. *Pieris brassicae* (great white butterfly) eradication annual report 2014/15 (Department of Conservation Annual Report No. R77017), Pieris brassicae eradication. Department of Conservation, Nelson, New Zealand.

Phillips, C., Brown, K., Green, C., Walker, G., Broome, K., Toft, R., Vander Lee, B., Shepherd, M., Bayley, S., Rees, J., 2014a. *Pieris brassicae* (great white butterfly) eradication annual report 2013/14 (Department of Conservation Annual Report). Nelson, New Zealand.

Phillips, C., Brown, K., Green, C., Walker, G., Broome, K., Vander Lee, B., King, M., 2013a. Great White Butterfly Interim Report Prepared for Ministry for Primary Industries External Technical Advisory Group, December 2013 (No. docDM-1307089). Department of Conservation, Nelson.

Phillips, C., Green, C., Walker, G., Broome, K., Brown, K., 2013b. Great White Butterfly Eradication Feasibility Assessment, November 2013. Department of Conservation, Nelson.

Phillips, C., Kean, J., 2013. Predicted seasonal occurrence of *Pieris brassicae* (great white butterfly) larvae at different New Zealand locations. (Report to Department of Conservation). AgResearch, Lincoln.

Phillips, C., Kean, J., van Koten, C., 2014b. Detection rates of *Pieris brassicae* eggs and larvae (No. RE400/2014/589), Using surveillance records to support eradication of great white butterfly from Nelson. AgResearch, Lincoln.

Phillips, C., Novoselov, M., Toft, R., 2015b. Ultraviolet reflectivity of *Pieris brassicae* wings and of materials used for *Pieris* lures (AgResearch Client Report).

Phillips, C., van Koten, C., 2014. What search radius around an infested property will optimise search efficiency for *Pieris brassicae*? (No. 884), Using surveillance records to support eradication of great white butterfly from Nelson. AgResearch, Lincoln.

Pluess, T., Jarošík, V., Pyšek, P., Cannon, R., Pergl, J., Breukers, A., Bacher, S., 2012. Which factors affect the success or failure of eradication campaigns against alien species? PloS one 7, e48157.

R Core Team, 2019. R: A language and environment for statistical computing. R Foundation for Statistical Computing, Vienna, Austria. URL https://www.R-project.org/.

Richards, N., Hardwick, S., Toft, R., Phillips, C., 2016. Enhancing parasitism of *Pieris brassicae* pupae by *Pteromalus puparum*. New Zealand Plant Protection 69, 126–132.

Richardson, E., Voice, D., 2010. Rapid Assessment Report on *Pieris brassicae* (Rapid Assessment No. IDC 2010 681 161). Biosecurity New Zealand.

Russell, J.C., Broome, K.G., 2016. Fifty years of rodent eradications in New Zealand: another decade of advances. New Zealand Journal of Ecology 40, 197–204.

Schulz, A.N., Lucardi, R.D., Marsico, T.D., 2019. Successful Invasions and Failed Biocontrol: The Role of Antagonistic Species Interactions. BioScience. https://doi.org/10.1093/biosci/biz075

Simberloff, D., 2009. We can eliminate invasions or live with them. Successful management projects. Biological Invasions 11, 149–157.

Simberloff, D., 2002. Today Tiritiri Matangi, tomorrow the world! Are we aiming too low in invasives control. Turning the tide: the eradication of invasive species 4–12.

Simberloff, D., Martin, J.-L., Genovesi, P., Maris, V., Wardle, D.A., Aronson, J., Courchamp, F., Galil, B., García-Berthou, E., Pascal, M., 2013. Impacts of biological invasions: what's what and the way forward. Trends in ecology & evolution 28, 58–66.

Spieth, H.R., Cordes, R., 2012. Geographic comparison of seasonal migration events of the large white butterfly, *Pieris brassicae*. Ecological Entomology 37, 439–445. https://doi.org/10.1111/j.1365-2311.2012.01385.x

Spieth, H.R., Pörschmann, U., Teiwes, C., 2011. The occurrence of summer diapause in the large white butterfly *Pieris brassicae* (Lepidoptera: Pieridae): A geographical perspective. Eur. J. Entomol 108, 377–384.

Spieth, H.R., Sauer, K.P., 1991. Quantitative measurement of photoperiods and its significance for the induction of diapause in *Pieris brassicae* (Lepidoptera, Pieridae). Journal of Insect Physiology 37, 231–238.

Stavenga, D., Arikawa, K., 2006. Evolution of color and vision of butterflies. Arthropod structure & development 35, 307–318.

Sullivan, T., Park, K., Manning, L., Twiddle, A., Brown, R., Suckling, D., 2014. Identification of semiochemicals in *Pieris rapae* and *P. brassicae* (Interim Progress Report). Plant and Food Research Ltd, Lincoln.

Tobin, P., Kean, J., Suckling, D., McCullough, D., Herms, D., Stringer, LD, 2014. Determinants of successful arthropod eradication programs. Biological Invasions 16, 401–414. https://doi.org/DOI 10.1007/s10530-013-0529-5

Toft, R., 2013. Great White Butterfly Eradication Annual Report 2012/13 (No. ENT-031). Entecol.

Toft, R., Brown, K., Courtney, S., Green, C., 2012. Proposed Eradication Strategy for Great White Cabbage Butterfly (*Pieris brassicae*). EntEcol.

Towns, D., Broome, K., Saunders, A., 2018. Ecological restoration on New Zealand islands: a history of shifting scales and paradigms. Australian Island Arks: Conservation, Management and Opportunities 205.

Townsend, A.J., de Lange, P.J., Duffy, C.A., Miskelly, C.M., Molloy, J., Norton, D.A., 2008. New Zealand threat classification system manual. Department of Conservation, Wellington 16, 2008–11.

Vitousek, P.M., D'Antonio, C.M., Loope, L.L., Rejmánek, M., Westbrooks, R., 1997. Introduced species: a significant component of human-caused global change. NZ J of Ecology 21, 1–16.

Vreysen, M., Robinson, A., Hendrichs, J., 2007. Area-wide control of insect pests: from research to field implementation. Springer Science & Business Media, Dordrecht, The Netherlands.

Walker, G, MacDonald, F., Wallis, R., Shaw, P., 2013. Management of the Great White Butterfly (Report for Sustainable Farming Fund, and Vegetables NZ No. 8929). Plant and Food Research Ltd, Mt Albert, Auckland.

Walker, GP, MacDonald, F., Wright, P., Connolly, P., 2013. Great White Butterfly Trap Improvements. Schedule 1: Improve visual lure and kill (IVLK) for *Pieris brassicae*, great white butterfly (GWB) (Report for Ministry for Primary Industries No. 8829), Client Project No. 16400. Plant and Food Research Ltd, Mt Albert, Auckland.

Walker, G., Shaw, P., Wallis, R., MacDonald, F., Harnett, D., 2014. Surveys of parasitism of larvae of *Pieris* species in Nelson 2013/14 (Draft summary for DOC GWB eradication project annual reporting 2013/14 No. 10205). Plant and Food Research Ltd, Mt Albert, Auckland.

Wardle, D.A., Bardgett, R.D., Callaway, R.M., Van Der Putten, W.H., 2011. Terrestrial ecosystem responses to species gains and losses. Science 332, 1273–1277. https://doi.org/10.1126/science.1197479.

Wickham, H., 2017. tidyverse: Easily Install and Load the 'Tidyverse'. R package version 1.2.1. https://CRAN.R-project.org/package=tidyverse.

Figure captions

Fig. 1. Map of Nelson and its environs with the *Pieris brassicae* eradication operational area shaded in blue. The red rectangle in the inset map indicates the position of the main map relative to the rest of New Zealand.

Fig. 2. Monthly *Pieris brassicae* detection rates from February 2013 to June 2016. Error bars show 95% binomial confidence intervals.

Fig. 3. Spatial distribution of *Pieris brassicae* from May 2010 to June 2016. Green markers show search locations where *P. brassicae* was not detected and red markers show locations where it was detected.

 Click here to access/download;Figure;fig_1.tiff

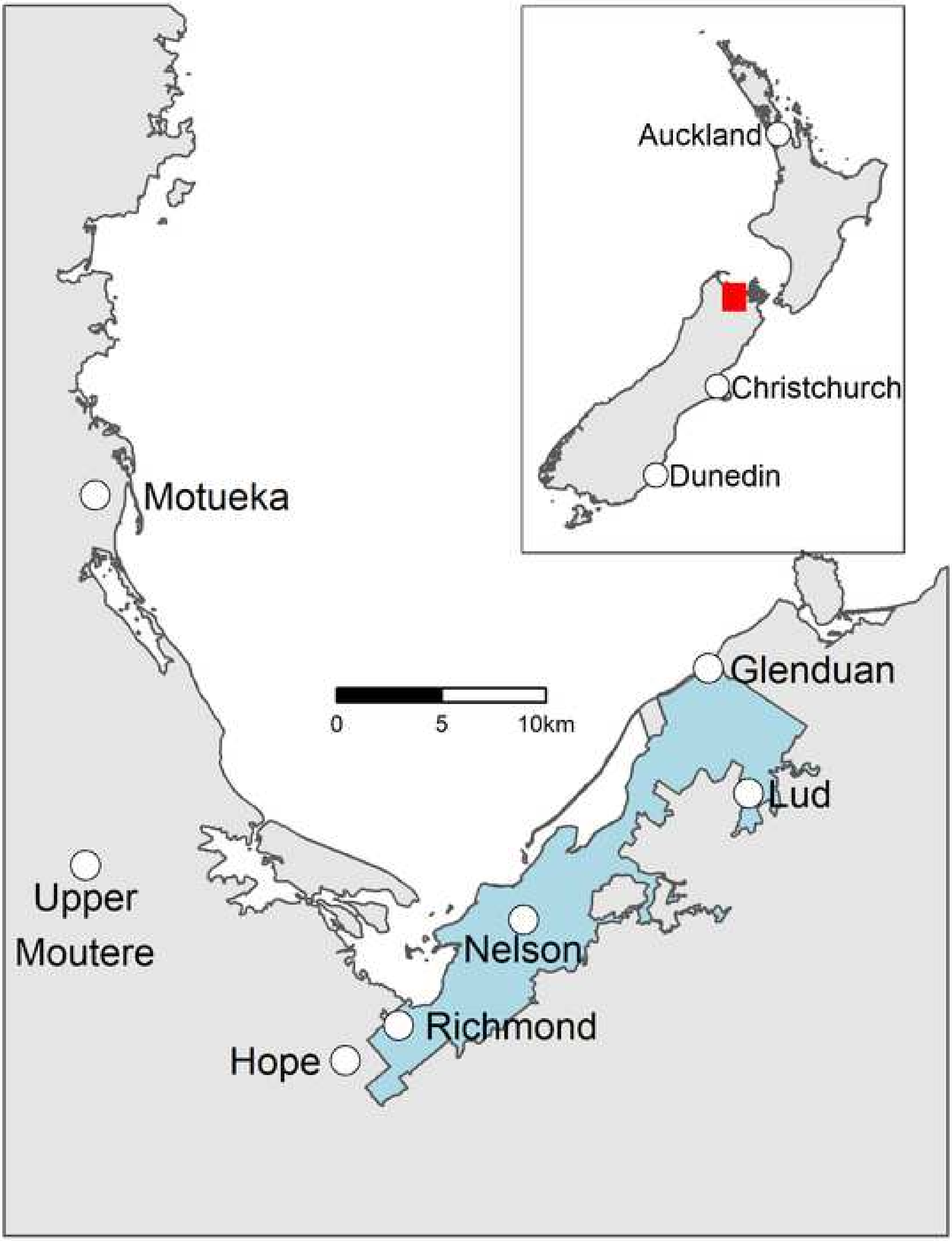

Figure 2

Click here to access/download;Figure;fig_2.tiff

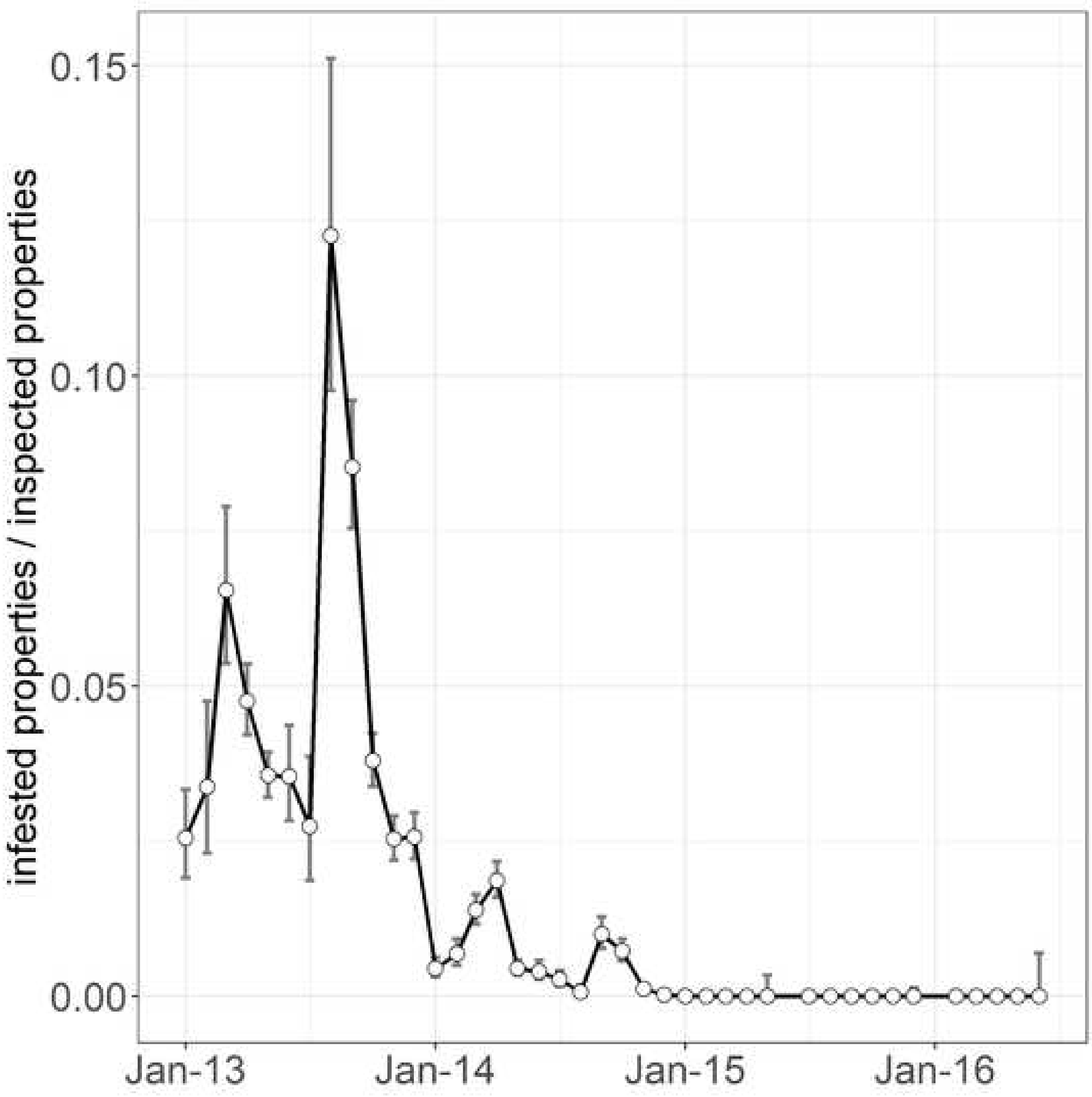

Figure 3

Click here to access/download;Figure;fig_3.tiff

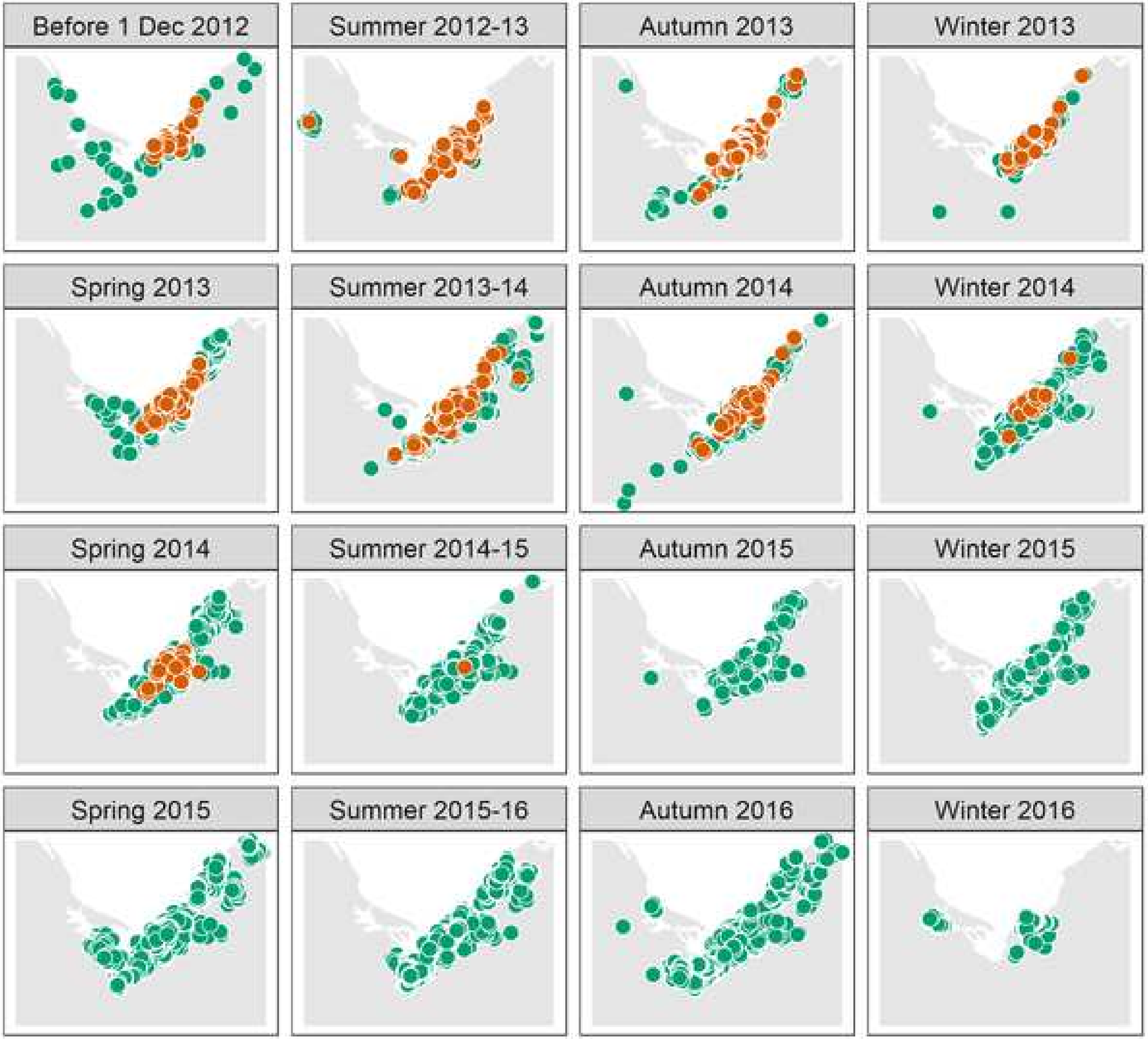

Click here to access/download
Supporting Information
additional_information_1.docx

AI 2

Click here to access/download
Supporting Information
additional_information_2.docx

Click here to access/download
Supporting Information
additional_information_3.docx
