# Supplementary material for: Eradicating the large white butterfly from New Zealand eliminates a threat to endemic Brassicaceae
Source: PLoS One. 2020 Aug 6;15(8):e0236791. doi: 10.1371/journal.pone.0236791 (PMC7410255; doi:10.1371/journal.pone.0236791)
Supplement: Attachment — Submitted filename: response_to_reviewers_2.docx [file pone.0236791.s006.docx]

**Eradicating large white butterfly from New Zealand eliminates a threat to endemic Brassicaceae**

**Responses to reviewers**

We have complied with the request to add our ethics statement to the beginning of the Methods section. However, please note that in order to maintain consistency with other parts of the manuscript, in the added text we have abbreviated ‘the New Zealand Department of Conservation‘ to ‘DOC’ and ‘Ministry for Primary Industries’ to ‘MPI’.
