# Supplementary material for: Eradicating the large white butterfly from New Zealand eliminates a threat to endemic Brassicaceae
Source: PLoS One. 2020 Aug 6;15(8):e0236791. doi: 10.1371/journal.pone.0236791 (PMC7410255; doi:10.1371/journal.pone.0236791)
Supplement: Attachment — Submitted filename: PONE-D-19-32860_R1_reviewer_SS.docx [file pone.0236791.s007.docx]

| Title: | Eradicating large white butterfly from New Zealand eliminates a threat to endemic *Brassicaceae* |
| --- | --- |
| Manuscript ID: | PONE-D-19-32860R1 |
| Authors: | Craig Phillips, Kerry Brown, Chris Green, Richard Toft, Graham Walker, Keith Broome |
| Manuscript Type: | Research Article |
| Journal: | PLoS ONE |

# Comments to the editor

As per my earlier communication, I disclosed that I at some point worked on this project. I, to the best of my knowledge objectively reviewed the manuscript as well as the authors’ response to the previous reviewers’ concerns. I believe the concerns were addressed adequately. Please feel free to contact me if you have any concerns or questions.

# Comments to the editor and corresponding Author

Since this is a second round review, I first went through the previous reviewers’ questions and the authors’ responses as well as the subsequent edit they made to the manuscript in response to the reviewers’ concerns. I would like to congratulate the previous reviewers on the critical and robust interrogation they made on the original manuscript. I am also intrigued by the detail at which the authors responded to each and every question. Even if it is quite difficult to judge on behalf of the original reviewers’ as they probably have a set way they expected concerns to be addressed, from my point of view I believe that the concerns raised are fully addressed.

I would here like to comment that I understand the reservations put forward by one of the previous reviewer that quantitative assessment of the measures taken to eradicate *Pieris brassicae* is lacking. However, I believe it is important to recognize that such eradication efforts are usually run by mandated government organizations whose primary goal is to eradicate the species; therefore, any activity that might be really interesting or important from future research point of view might not necessarily be prioritized. Yet, since the eradication was a success even if it is hard to quantify which specific measure contributed how much towards the success, it is still a very important source where future researchers and managers alike can find detailed prescriptions of how each measure was implemented. And since the sum of these different measures, save the natural enemies and terrain, resulted in a successful eradication the article already would make one tried and tested integrated eradication management system available for end-users.

I have the following two recommendations for the authors

**Recommendation 1**

Mandated bodies usually take recommendations from researchers to optimize the eradication but not necessarily take steps to provide data that can be used to assess the efficacy of taken measure with the same rigor they carry out the eradication itself. In this regard, I believe it is appropriate for the authors to insert a recommendation for biostatisticians/pest risk modelers/population researchers/entomologists or others in a similar field to come up with a new set of - or modified indicators or parameters that can easily be measured by eradication campaigns. This will help quantitatively assessing the exercise without taxing the body who is carrying out the eradication too much time and effort for collecting the necessary data.

**Recommendation 2** ( this one is if the authors feel inclined and if they think it is not out of the scope of their paper)

As I mentioned a couple of times above, there is not enough information on details of how each of the measures taken contribute towards the final success. It is usually very difficult to attain such detail as such eradications are often carried out under limited resources and under government oversight making it difficult, to collect and analyze data needed for such analysis.

In short, it is not run like an experiment rather it is a job that needs to be done. However, I believe there is still a case to be made to such bodies that the more of such information they collect even if costly, it will in the end make overall eradication efforts economically sound. Therefore, It will be beneficial for future eradication endeavors if the authors recommend in their paper that MPI/DoC or any mandated body that might carry out such eradications to make room in their program to assess the benefits of each specific measure within their integrated eradication system. Such data is elemental in building bio-economic eradication models that can optimize success both in the economic and biological sense. Although, in such cases when endangered species are involved money might not be an issue, overall if there is data that can optimize eradication techniques and drive down cost. The decision to manage rather than eradicate harmful invasive species might have a different outcome.

# Line by line (minor)

L55: I believe in some ways the good outcome of the eradication effort is in some implicit way helped by the good system-wide understanding of best biosecurity practices in NZ. By that, I mean the increased awareness regarding biosecurity in New Zealand among the Government, Researchers and Citizens alike. It is important to give some reference besides the *P. brassicae* being listed as Unwanted Organism, that it has also been identified as a possible threat and invader by New Zealander researchers. For example Worner and Gevrey (2006) predicted its possible invasion in 2006.

L551: the use of a colon here rather signaled a list but what followed was two items separated by a semi colon. Better to remove the colon and continue with “allowed…” and replace the semi colon with a comma on L552. It will make it easy to read the paragraph.

**Reference:** Worner, S., & Gevrey, M. (2006). Modelling global insect pest species assemblages to determine risk of invasion. *Journal of Applied Ecology, 43*(5), 858-867.
