# Supplementary material for: Eradicating the large white butterfly from New Zealand eliminates a threat to endemic Brassicaceae
Source: PLoS One. 2020 Aug 6;15(8):e0236791. doi: 10.1371/journal.pone.0236791 (PMC7410255; doi:10.1371/journal.pone.0236791)
Supplement: Attachment — Submitted filename: response_to_reviewers_3.docx [file pone.0236791.s008.docx]

Reviewer #3

a. *Discussion … needs to be compacted and start with an initial paragraph summarizing the main findings and pointing to the principal issues that will be discussed in what follows. In its current form, the authors jump straight into discussing details.*

Authors: We have moved the first paragraph of the Discussion to the end of the Results, and rewritten the first paragraph of the Discussion in the manner suggested by the reviewer. (This is repeated in point ‘o’ below.)

b. *I also believe that even though Pieris brassicae is a broadly known species, the expert taxonomist that confirmed the identity of the specimens needs to be acknowledged, the place where voucher specimens are housed needs to be specified,*

Authors: On lines 71-73 we have added a reference that describes the process by which P. brassicae was formally identified by MPI and provided the name of a NZ lepidopterist who assisted MPI.

Further author comments not added to the manuscript: We searched the NZ Arthropod Collection’s on-line database (1-Jun-2020) and found no records of P. brassicae voucher specimens. We note that the diagnosis was subsequently supported by independent DNA sequencing in a study cited in the manuscript ([61] Hiszczynska-Sawicka & Phillips 2014).

c. *and further discussion on the possible origin on the invading “founders” based on the genetic studies performed is needed.*

Authors: We agree this is an interesting question, which was properly examined in a paper cited in the ‘Active surveillance’ section of the Results (“…analysing genetic variation in the mitochondrial COI gene of all detected specimens [61].”). That paper is freely available on-line and gives BOLD and GenBank accession numbers for numerous *P. brassicae*, including for specimens from New Zealand’s (NZ) population. However, the aim of the present manuscript is to provide an overview of how *P. brassicae* was eradicated, and possible non- NZ geographic sources of NZ’s *P. brassicae* population are irrelevant to this aim. No change made.

d. *L. 1 – Tittle. Is not a “the” missing after “Eradicating”. That is, “Eradicating the large white butterfly …..*

Authors: ‘the’ inserted.

e. *L. 34 – Abstract. Add “worldwide” in the end. That is, “This is the first species of butterfly ever to have been eradicated worldwide”.*

Authors: ‘worldwide’ added.

f. *L. 36-37. I do not believe that the keywords chosen by the authors are the ideal ones. In my opinion, the following words would be much better suited: eradication, Pieris brassicae, Lepidoptera, New Zealand, invasion biology, biosecurity, endemic flora conservation.*

Authors: We’re grateful for the suggestions and have edited the key words.

g. *L. 76 – Introduction. Here and many places elsewhere in manuscript (e.g., lines 305, 472, 483, 710, 726) numbers are used that should be spelled out in words. The grammatical rule indicates that any number smaller that 10 needs to be written in words not numbers. Here (L. 76) it reads “….. to Nelson 2 years after ….”. Believe it should be “two years”. Leave this to the editor as I do not know the specific rules of PLoS One.*

Authors: We’ve only used numerals less than 10 when they precede a unit (e.g. year), which we believe is the correct style. No change made.

h. *L. 118 – Methods. It would be very useful for the reader to have access to a summary figure with a clear flow chart detailing the most important strategies followed during the entire eradication program from start to end. A lot of actions were implemented, and the reader can get overwhelmed with so much detail. Therefore, a tidy, well thought out/designed flow chart leading the reader step by step as to the critical strategies followed in the “command room” by the chief program manager, would greatly simplify reading/perusing over all the details.*

Authors: We attempted to produce a clear summary figure as suggested by the referee but the resulting draft diagrams were too complex to be helpful. We decided that a Table would better meet the need indicated by the referee and have added this to the manuscript as Table 1.

i. *Somewhere in this section, the authors need to specify who formally/taxonomically identified Pieris brassicae, Cotesia glomerata, Pteromalus puparum, indicate in which officially recognized insect collection are the voucher specimens saved, and also where is the genetic material saved for future verifications (i.e., mitochondrial COI gene sequences) in the case of Pieris brassicae. Independent of the fact that P. brassicae is a well-known insect, these types of formalities cannot be overridden.*

Authors: We’ve provided information about the initial identification of *P. brassicae* in the Introduction (L71-73) – see point ‘b’ above. However, we consider details of how *C. glomerata* and *Pt. puparum* were identified superfluous to this manuscript because work involving them is only briefly mentioned. Any readers who are particularly interested in the work with *Pt. puparum* can either read cited paper [53] or contact the authors (Richards et al 2016), and those who want more details of *C. glomerata* can contact the authors of cited reports [51] and [52] (Walker, who is also an author of the present manuscript). Similarly, readers interested in COI sequences for *P. brassicae* can read cited article [61] or contact its authors (Hiszczynska-Sawicka & Phillips 2014).

j. *L. 435/436. Explain if such a broad range (32-52% and 5-25%) did not entail the risk of undetected eggs/larvae. This is a minor point as the end result was eradication …*

Authors: We do not understand this suggestion. The text in question explains that some eggs and larvae indeed remained undetected, and the proportion undetected declined as search methods were refined and insecticide use began. No change made.

k. *L. 466. Capitalize N in “nasturtium“.*

Authors: We disagree nasturtium should be capitalised. It is a common name and is neither a genus, nor named after a person or place. No change made.

l. *L. 476-479. A bit confusing. Was every telephone report (direct or toll-free) later physically confirmed by an expert? Was the ID of all specimens verified?*

Authors: We disagree this is confusing. It is clearly stated in the Methods that “*Reports were immediately conveyed to DOC, which responded within 48 hours, usually visiting the properties for verification*” (‘Passive surveillance’ section), and that *field staff were trained to identify* P. brassicae (‘Field staff’ section). No change made.

m. *L. 481-485. Any idea as to what the origin of the invading population was? The authors mention that they kept mitochondrial COI gene information on all specimens sampled. If so, this information could be matched with data kept at official repositories (e.g., gene bank) and obtain an idea with respect of the origin of the invaders. This information would be useful to avoid reinvasions, and to strengthen pathway regulations.*

Authors: We addressed this previously in point ‘c’.

n. *L. 563-605 are repeated in Lines 607-48.*

Authors: We could not find the repetition mentioned. No change made.

o. *L. 563 – Discussion. This section needs to be started with a paragraph summarizing the most significant results of the previous section (Results), outlining in the end the approach that will be followed in discussing these relevant findings. Currently, the first paragraph of the Discussion (Lines 564-576), leads the reader too abruptly into details that do not provide an overview of the relevant findings.*

Authors: We have moved the first paragraph of the Discussion to the end of the Results, and have rewritten the first paragraph of the Discussion in the manner suggested by the reviewer.

p. *In addition, I feel the entire Discussion section could be tightened/compacted and better organized.*

Authors: We comprehensively restructured the Discussion so that it follows the structure described in the Discussion’s first paragraph. We also removed some superfluous text.

**Reviewer #4**

a. *Recommendation 1: Mandated bodies usually take recommendations from researchers to optimize the eradication but not necessarily take steps to provide data that can be used to assess the efficacy of taken measure with the same rigor they carry out the eradication itself. In this regard, I believe it is appropriate for the authors to insert a recommendation for biostatisticians/pest risk modelers/population researchers/entomologists or others in a similar field to come up with a new set of - or modified indicators or parameters that can easily be measured by eradication campaigns. This will help quantitatively assessing the exercise without taxing the body who is carrying out the eradication too much time and effort for collecting the necessary data.*

Authors: We consider the suggested recommendation infeasible and unlikely to be followed for the following reasons:

1. It would of course have been interesting to know the precise efficacy of each *P. brassicae* control method, but would such information have caused the *P. brassicae* program to change? Probably not – because if a control method was affordable, acceptable, practical and had a non zero probability of limiting *P. brassicae*, it was implemented irrespective of whether it might kill 10 *P. brassicae* individuals or 10000. This helped to ‘put every pest individual at risk of control at some stage of its development’, and ‘prevent pest immigration and emigration’ (two criteria of Phillips et al. 2019 [33]). It also provided managers with a range of options for deploying staff according to availability, skills, time of year and current priorities. As noted by the reviewer, however, modeling and statistical analyses were frequently used to help optisimise the impact of each control method. We also note that the program did evaluate the efficacy of control measures to the extent possible (see results for ‘Active Surveillance’ that estimated the efficacy of searching and applying insecticide, and the benefit of using the UV lure).
2. The difficulty of accurately quantifying the independent effects of different control methods on pest populations is well recognised. Even in integrated pest management, where properly designed experiments with controls are feasible and widely practiced, researchers can seldom accurately quantify the independent effects of different controls (eg. natural enemies, pesticides, resistant plant cultivars, cultural controls) on pest population densities/sizes. The numerous impediments to doing so include: An absence of absolute unbiased field sampling methods for most organisms; spatial, temporal, climatic and genetic variability; difficulty of removing or controlling all confounded factors; and strong and often poorly understood interactions between system components. We suggest a more appropriate place for the reviewer’s recommendation would be in the integrated pest management literature where proper experimentation is readily achievable and distractions from the primary goal of eradication are more tenable. Eradication programs already draw strongly from pest management research, and would readily adopt pest management advances made in this area.
b. *Recommendation 2 ( this one is if the authors feel inclined and if they think it is not out of the scope of their paper). As I mentioned a couple of times above, there is not enough information on details of how each of the measures taken contribute towards the final success. It is usually very difficult to attain such detail as such eradications are often carried out under limited resources and under government oversight making it difficult, to collect and analyze data needed for such analysis.*

*In short, it is not run like an experiment rather it is a job that needs to be done. However, I believe there is still a case to be made to such bodies that the more of such information they collect even if costly, it will in the end make overall eradication efforts economically sound. Therefore, It will be beneficial for future eradication endeavors if the authors recommend in their paper that MPI/DoC or any mandated body that might carry out such eradications to make room in their program to assess the benefits of each specific measure within their integrated eradication system. Such data is elemental in building bio-economic eradication models that can optimize success both in the economic and biological sense. Although, in such cases when endangered species are involved money might not be an issue, overall if there is data that can optimize eradication techniques and drive down cost. The decision to manage rather than eradicate harmful invasive species might have a different outcome.*

Authors: The reviewer’s Recommendation 2 is essentially the same as their Recommendation 1 except the former is directed at government biosecurity authorities and the latter at researchers. Please see our response to the first recommendation.

c. *L55: I believe in some ways the good outcome of the eradication effort is in some implicit way helped by the good system-wide understanding of best biosecurity practices in NZ. By that, I mean the increased awareness regarding biosecurity in New Zealand among the Government, Researchers and Citizens alike. It is important to give some reference besides the P. brassicae being listed as Unwanted Organism, that it has also been identified as a possible threat and invader by New Zealander researchers. For example Worner and Gevrey (2006) predicted its possible invasion in 2006.*

Authors: We thank the reviewer for reminding us of Worner & Gevrey’s predictions, and have cited their study at the end of the 2^nd^ paragraph of the Introduction.

d. *L551: the use of a colon here rather signaled a list but what followed was two items separated by a semi colon. Better to remove the colon and continue with “allowed…” and replace the semi colon with a comma on L552. It will make it easy to read the paragraph.*

Authors: Change made as suggested.
